# Association of Digital Health Interventions With Maternal and Neonatal Outcomes: Systematic Review and Meta-Analysis

**DOI:** 10.2196/66580

**Published:** 2025-03-14

**Authors:** Jianing Wang, Nu Tang, Congcong Jin, Jianxue Yang, Xiangpeng Zheng, Qiujing Jiang, Shengping Li, Nian Xiao, Xiaojun Zhou

**Affiliations:** 1 Department of Maternal and Child Information Management Women and Children's Hospital of Chongqing Medical University Chonqing China; 2 Community Health Care Office Women and Children's Hospital of Chongqing Medical University Chongqing China; 3 Pregnancy Health Center Women and Children's Hospital of Chongqing Medical University Chongqing China; 4 Department of Maternal and Child Health Chongqing Municipal Health Commission Chongqing China; 5 Department of Health Education Women and Children's Hospital of Chongqing Medical University Chongqing China; 6 Department of Child Healthcare Women and Children's Hospital of Chongqing Medical University Chongqing China; 7 Department of Equipment Management Women and Children's Hospital of Chongqing Medical University Chongqing China

**Keywords:** digital health, telemedicine, telehealth, mobile health, mHealth, mobile phone, intervention, meta-analysis, pregnant women, systematic review

## Abstract

**Background:**

Gestational weight gain (GWG) is crucial to maternal and neonatal health, yet many women fail to meet recommended guidelines, increasing the risk of complications. Digital health interventions offer promising solutions, but their effectiveness remains uncertain. This study evaluates the impact of such interventions on GWG and other maternal and neonatal outcomes.

**Objective:**

This study aimed to investigate the effect of digital health interventions among pregnant women and newborns.

**Methods:**

A total of 2 independent researchers performed electronic literature searches in the PubMed, Embase, Web of Science, and Cochrane Library databases to identify eligible studies published from their inception until February 2024; an updated search was conducted in August 2024. The studies included randomized controlled trials (RCTs) related to maternal and neonatal clinical outcomes. The Revised Cochrane risk-of-bias tool for randomized trials was used to examine the risk of publication bias. Stata (version 15.1; StataCorp) was used to analyze the data.

**Results:**

We incorporated 42 pertinent RCTs involving 148,866 participants. In comparison to the routine care group, GWG was markedly reduced in the intervention group (standardized mean difference–0.19, 95% CI –0.25 to –0.13; *P<*.001). A significant reduction was observed in the proportion of women with excessive weight gain (odds ratio [OR] 0.79, 95% CI 0.69-0.91; *P*=.001), along with an increase in the proportion of women with adequate weight gain (OR 1.33, 95% CI 1.10-1.64; *P=.*003). Although no significant difference was reported for the proportion of individuals below standardized weight gain, there is a significant reduction in the risk of miscarriage (OR 0.66, 95% CI 0.46-0.95; *P=.*03), preterm birth (OR 0.8, 95% CI 0.75-0.86; *P*<.001), as well as complex neonatal outcomes (OR 0.93, 95% CI 0.87-0.99; *P*=.02). Other maternal and fetal outcomes were not significantly different between the 2 groups (all *P*>.05).

**Conclusions:**

The findings corroborate our hypothesis that digitally facilitated health care can enhance certain facets of maternal and neonatal outcomes, particularly by mitigating excessive weight and maintaining individuals within a reasonable weight gain range. Therefore, encouraging women to join the digital health team sounds feasible and helpful.

**Trial Registration:**

PROSPERO CRD42024564331; https://tinyurl.com/5n6bshjt

## Introduction

Pregnancy is a unique physiological phase marked by significant physical, psychological, and behavioral changes that impact maternal and neonatal outcomes [1]. A key aspect of pregnancy is gestational weight gain (GWG), which plays a crucial role in maternal and infant health. However, studies indicate many women fail to meet the recommended GWG guidelines. In 2018, only 28%, 31%, and 32% of women in the United States, Europe, and Asia achieved the recommended weight gain during pregnancy [2]. This issue is even more prevalent in low- and middle-income countries. A 2023 study across 24 countries found that 55% (65,505/118,207) of participants experienced inadequate GWG, 23% (26,746/118,207) gained excessive weight, and only 22% (25,956/118,207) adhered to the recommended guidelines [3].

Maternal weight gain has a profound impact on pregnancy outcomes, including gestational complications, infant mortality, and long-term health for both mother and child [4]. Excessive GWG is linked to higher risks of complications such as large for gestational age (LGA), macrosomia, cesarean delivery, and postpartum weight retention [5-7]. On the other hand, insufficient GWG is associated with increased risks of miscarriage, infants who are small for gestational age (SGA), low birth weight, and preterm birth [8-10]. Therefore, promoting healthy gestational weight gain is crucial in reducing pregnancy complications and minimizing the risks of maternal and neonatal morbidity and mortality.

Digital health interventions, including applications, websites, digital programs, and other smart devices, have gained significant attention for their potential to enhance physical and mental well-being, particularly in low-resource settings such as Africa and South Asia [11]. Telemedicine involves using telecommunications technology to deliver clinical health care remotely, enabling health care providers to diagnose, treat, and monitor patients from a distance. Telehealth is a broader concept encompassing telemedicine and additional services, such as health education, disease prevention, and remote monitoring. Mobile health (mHealth) refers explicitly to using mobile devices like smartphones and tablets to deliver health care services, track health conditions, and promote healthy behaviors [12]. While these technologies overlap, each serves a distinct purpose, and together, they form key components of modern health care interventions.

Evidence suggests that technology-mediated interventions can be as effective or superior to routine care in improving maternal and neonatal health outcomes [13]. For example, a meta-analysis found that digital health interventions for mothers with gestational diabetes improved self-care, leading to better weight and glycemic control and lower rates of macrosomia and cesarean deliveries [14]. Another meta-analysis, combining data from 21 randomized controlled trials (RCTs) and controlled clinical trials, reported that web-based interventions significantly increased the likelihood of vaginal delivery while reducing emergency cesarean sections and neonatal complications. However, no improvements in glucose profiles were observed [15]. Furthermore, a study by He et al [16] demonstrated that mHealth interventions significantly decreased the incidence of gestational diabetes, preterm births, and macrosomia in pregnant women with overweight or obesity. In addition, participants in the intervention group gained 1.12 kg less than those in the routine care group [16]. However, some studies have contradicted these findings, reporting no significant impact of telemedicine on maternal or neonatal outcomes [17-19].

The contradictory findings underscore the need for further research into the effectiveness of digital health interventions, especially to evaluate their impact on pregnant women with varying risk profiles and refine strategies to improve maternal and neonatal health outcomes. To our knowledge, this is the first meta-analysis to broaden the participant scope, including not only high-risk groups, such as those with gestational diabetes, overweight, or obesity, but also low-risk or nonspecific pregnant women. This review aims to systematically evaluate studies investigating the impact of digital health interventions on maternal health outcomes, including gestational weight management and neonatal health outcomes in pregnant women.

## Methods

### Overview

The study protocol was preregistered in PROSPERO (CRD42024564331). The manuscript was structured following the PRISMA (Preferred Reporting Items for Systematic Reviews and Meta-Analyses) checklist [20], and the checklist is presented in Multimedia Appendix 1.

### Search Strategy

The initial comprehensive literature search for this meta-analysis was conducted in February 2024 and updated in August 2024 to capture any newly published studies. The search spanned 4 major English-language databases: PubMed, Embase, Cochrane Library, and Web of Science. Keywords derived from relevant articles were used, including terms such as telemetry, digital health, e-consultation, telemonitoring, smartphone technology, online communication, and digital health technology. Details of the search strategy are provided in Multimedia Appendix 2.

### Inclusion and Exclusion Criteria

Eligible studies were required to be RCTs published in English, focusing on pregnant women aged 18 years and older. Digital health interventions, such as phone calls, text messages, and interactive apps (eg, YouTube, Twitter, and WeChat), were implemented in the intervention group, while the control group received standard care. The studies evaluated either maternal outcomes, such as GWG and pregnancy complications, or neonatal outcomes, such as preterm birth and SGA, defined as a birth weight at or below the 10th percentile for gestational age.

Studies were excluded if they did not include a control group, involved both intervention and control groups receiving digitally mediated treatments, failed to report the desired outcomes, measured them only postpartum, or lacked accessible full-text articles or usable data.

### Study Selection and Data Extraction

Following the removal of duplicates, 2 researchers (JW and NT) independently screened the remaining articles by evaluating their titles and abstracts, excluding those that were irrelevant. The full texts of studies identified as potentially relevant were retrieved and further assessed to determine their eligibility for inclusion. In cases where discrepancies arose between the two researchers, these were resolved through discussion or, if necessary, by consulting a third researcher to achieve consensus.

For articles selected for further analysis, JW and NT used a standardized data extraction worksheet developed in Microsoft Excel 2016. The extracted data encompassed key study characteristics, including the year of publication, authors, country, sample size, inclusion and exclusion criteria, maternal and neonatal health outcomes, gestational age at enrollment, prepregnancy maternal BMI, type of digital applications, specific interventions, control measures, duration of intervention, high-risk factors of participants, and the effects of digital care on maternal and neonatal health. Any disagreements during the data extraction process were resolved through iterative discussions between the authors until a consensus was reached.

### Evaluation of the Methodological Quality of the Studies

The bias of the RCTs included in this meta-analysis was evaluated using the Revised Cochrane risk-of-bias tool for randomized trials (RoB 2) [21]. JW and NT independently assessed the risk of bias for each included study. Discrepancies between the two reviewers were resolved through iterative discussions, revisiting study details, and assessment criteria to reach a consensus. If disagreements persisted, a third reviewer provided an independent evaluation, with the final decision based on majority agreement.

### Data Analysis

Data analysis for the meta-analysis was performed using Stata (version 15.1; StataCorp). Effect sizes were calculated and presented as forest plots to facilitate quantitative synthesis. Standardized mean differences (SMDs) were used for continuous variables, while odds ratios (ORs) were applied for dichotomous outcomes. The choice between fixed-effect and random-effects models was determined by the level of heterogeneity, with *I*² values above 50% indicating substantial heterogeneity; a random-effects model was used for *I*²>50%, while *I*²≤50% warranted a fixed-effect model. A *P* value of <.05 was considered statistically significant. To assess the robustness of the synthesized results, sensitivity analyses were conducted by sequentially excluding each study and reanalyzing the data, as well as by restricting the analysis to studies with a low risk of bias. To assess a potential publication bias, funnel plots were used, and the Egger regression test was used to calculate the publication bias (Multimedia Appendix 3).

## Results

### Search Results

**Figure 1:**
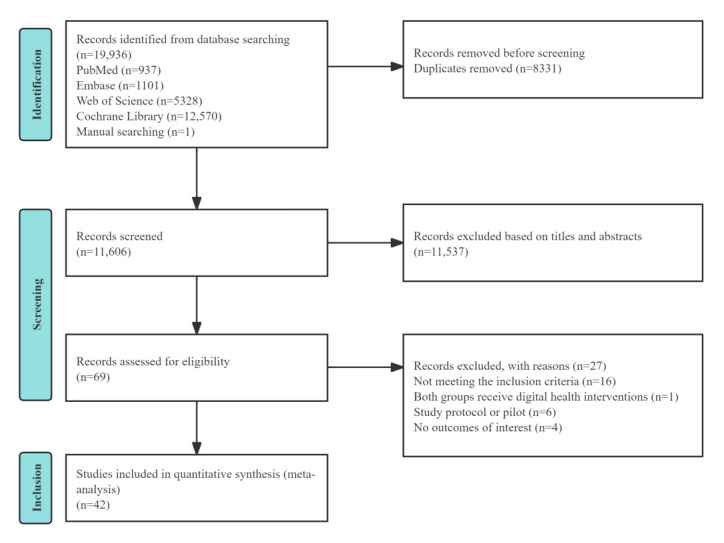
PRISMA (Preferred Reporting Items for Systematic Reviews and Meta-Analyses) flow diagram of the literature screening and selection process.

A total of 19,936 studies were retrieved from the 4 databases, and 1 additional article was identified through a manual search. After removing duplicates, 11,606 studies remained for further evaluation. Titles and abstracts were screened, and 69 studies were deemed relevant. Following a detailed review of the full texts, 42 RCTs met the inclusion criteria and were incorporated into the meta-analysis. Figure 1 shows the complete screening process.

### Study Characteristics

This meta-analysis includes data from 42 RCTs involving a total of 148,866 participants. Its primary focus is to evaluate the impact of digital health interventions on maternal and neonatal outcomes, particularly among populations with gestational diabetes mellitus (GDM) and other high-risk pregnancy factors. The studies, conducted between 2007 and 2024, were geographically distributed across Asia (13/42 studies, 31%), Europe (13/42 studies, 31%), North America (9/42 studies, 21%), Australasia (6/42 studies, 14%), and Africa (1/42 study, 3%). The majority of studies were published in the United States (9/42 studies, 21%), Australia (6/42 studies, 14%), and China (6/42 studies, 14%).

Digital health interventions used three primary delivery modalities: (1) mobile devices, including smartphones and tablets; (2) website-based platforms; and (3) mobile apps incorporating social software (eg, Facebook [Meta Platforms], Zoom [Zoom Communications, and WeChat [Tencent Holdings Limited]) and other digital health tools. Table 1 presents the detailed characteristics of the 42 RCTs [22-63].

**Table 1:** The characteristics of included studies.

Author, year	Country	Type of digital health	Duration of intervention	Sample size, n			High-risk factors
				IG^a^	CG^b^		
Homko et al [22], 2007	United States	Internet	To birth	32	25	GDM^c^	
Pérez-Ferre et al [23], 2010	Spain	Mobile phone	To birth	49	48	GDM	
Phelan et al [24], 2011	United States	Telephone	To birth	201	200	—^d^	
Homko et al [25], 2012	United States	Internet	To birth	40	40	GDM	
Given et al [26], 2015	United Kingdom	Internet	To birth	24	26	GDM	
Durnwald et al [27], 2016	United States	Telephone	To birth	49	52	GDM	
Herring et al [28], 2016	United States	SMS text message and telephone	To birth	33	33	Overweight or obesity	
Smith et al [29], 2016	United States	Internet	To birth	24	21	—	
Zairina et al [30], 2016	Australia	Mobile phone	To birth	36	36	Asthma	
Willcox et al [31], 2017	Australia	Telephone and internet	To birth	45	46	—	
Sagedal et al [32], 2017	Norway	Mobile phone	To 36 weeks gestation	296	295	—	
Kennedy et al [33], 2018	Ireland	Internet	To birth	125	125	—	
Kennelly et al [34], 2018	Ireland and the Netherlands	Mobile phone	To birth	278	287	GDM	
Mackillop et al [35], 2018	United Kingdom	Mobile phone	To birth	101	102	GDM	
Miremberg et al [36], 2018	Israel	Mobile phone	To birth	60	60	GDM	
Patel et al [37], 2018	India	SMS text message and telephone	6 months after delivery	519	518	—	
Rasekaba et al [38], 2018	Australia	Internet	To birth	61	34	GDM	
Al-Ofi et al [39], 2019	Saudi Arabia	Telemonitoring device and SMS text message	6 weeks after delivery	27	30	GDM	
Borgen et al [40], 2019	Norway	Mobile phone	To birth	115	123	GDM	
Carolan-Olah and Sayakhot [41], 2019	Australia	Internet	To birth	52	58	GDM	
Guo et al [42], 2019	China	Mobile phone	To birth	64	60	GDM	
Sung et al [43], 2019	South Korea	Mobile phone	To birth	11	10	GDM	
Butler Tobah et al [44], 2019	United States	Telemonitoring device and telephone	To birth	150	150	—	
Ferrara et al [45], 2020	United States	Telephone	To 38 weeks gestation	199	195	Overweight or obesity	
Huang et al [46], 2020	Australia	Internet	12 weeks	30	27	—	
Tomyabatra [47], 2020	Thailand	Mobile phone	To birth	432	400	—	
Ding et al [48], 2021	China	Mobile phone	To birth	104	111	Overweight or obesity	
LeBlanc et al [49], 2021	United States	Telephone and internet	To birth	89	80	Overweight or obesity	
Sandborg et al [50], 2021	Sweden	Mobile phone	6 months	152	153	—	
Su et al [51], 2021	China	Internet	6 months	56	56	GDM	
Sun and Lingying [52], 2021	China	Mobile phone	To birth	40	40	GDM	
Tian et al [53], 2021	China	Mobile phone	To birth	133	136	GDM	
Yew et al [54], 2021	Singapore	Telemedicine device and telephone	To birth	170	170	GDM	
Gonzalez-Plaza et al [55], 2022	Spain	Mobile phone	To birth	78	72	Obesity	
Uria-Minguito et al [56], 2022	Spain	Internet	To 38-39 weeks gestation	102	101	—	
Bekker et al [57], 2023	Netherlands	Telemonitoring device and telephone	To birth	100	100	—	
Munda et al [58], 2023	Slovenia	Telemedicine device and video conferencing system	To birth	53	52	GDM	
Sharma et al [59], 2023	India	Mobile phone	To birth	65	66	—	
Skalecki et al [60], 2023	Australia	Telemonitoring device	To birth	13,771	12,628	—	
Wakwoya et al [61], 2023	Ethiopia	SMS text message and telephone	To birth	163	163	—	
Téoule et al [62], 2024	Germany	Mobile phone	To birth	49	48	—	
Wang et al [63], 2024	China	Mobile phone	To birth	29	29	—	

^a^IG: intervention group.

^b^CG: control group.

^c^GDM: gestational diabetes mellitus.

^d^Not applicable.

### Quality Assessment Results of the Studies

**Figure 2:**
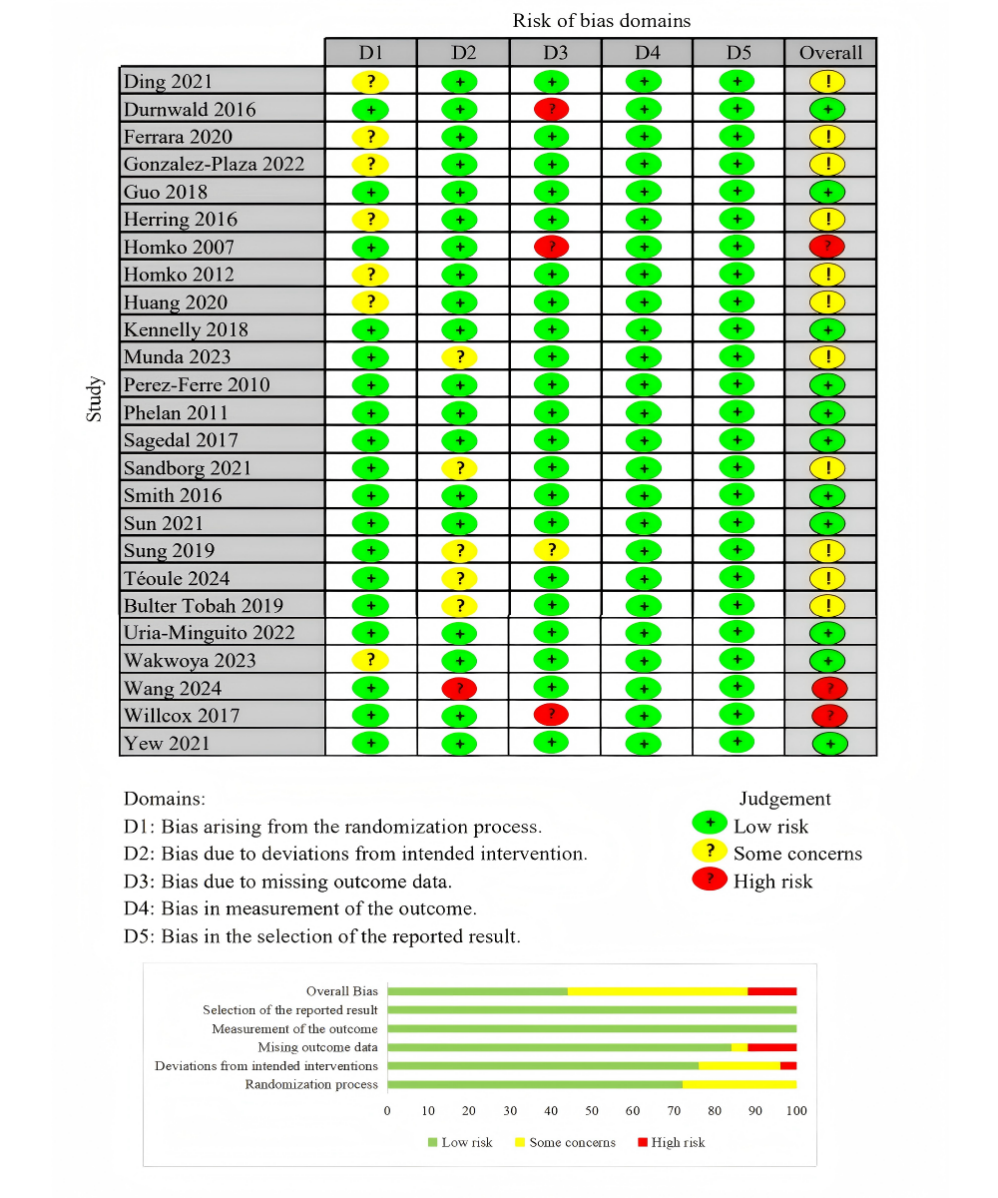
Risk-of-bias domains. ROB-2. RoB 2: Revised Cochrane risk-of-bias tool for randomized trials.

A total of 25 RCTs included in this review, focusing on GWG, were evaluated using the RoB 2 tool. Among these, 11 studies were determined to have a low risk of bias, 3 were identified as high risk, and 11 presented some concerns regarding potential bias. Studies that focused on secondary outcomes, such as neonatal health, were not included in this assessment, as the RoB 2 evaluation was specifically applied to studies addressing the primary outcome of GWG. Figure 2 [22-25,27-29,31,32,34,42-46,48,50,52,54-56,58,61-63] shows the risk-of-bias assessment.

### Meta-Analysis Results

#### Effect on GWG

**Figure 3:**
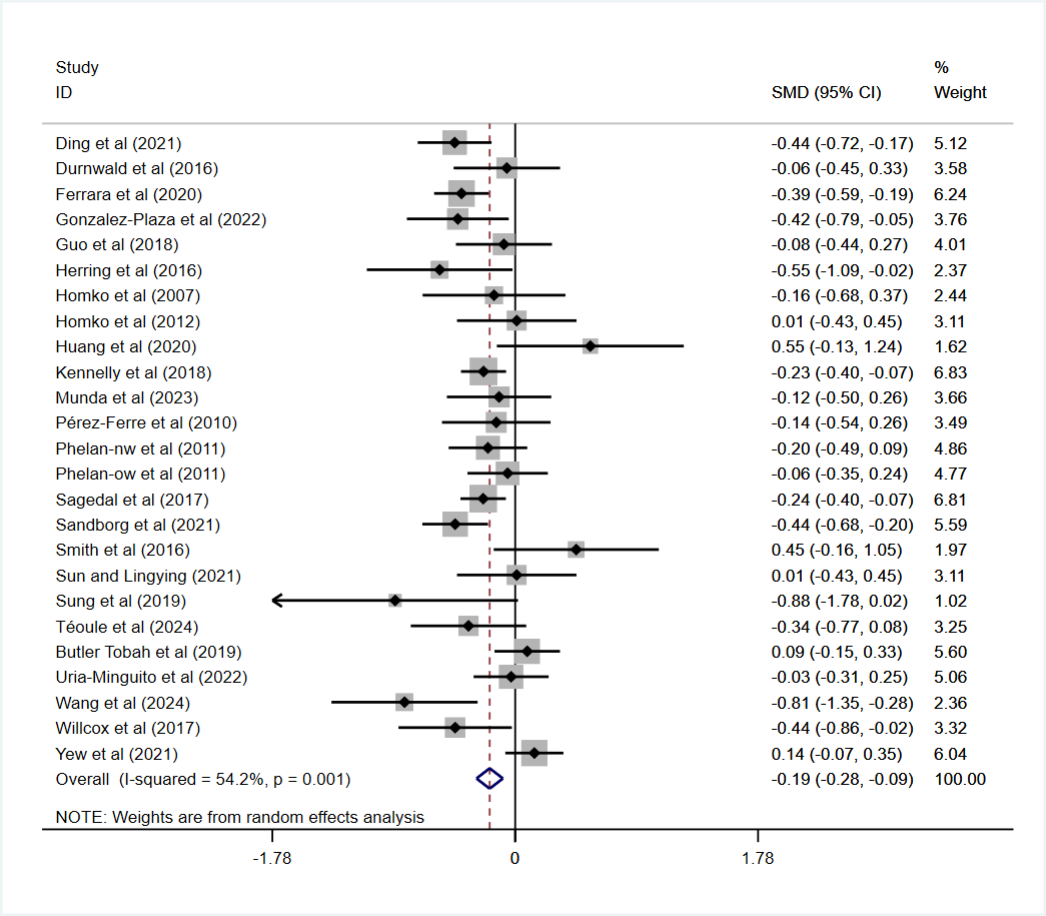
Effect on gestational weight gain. SMD: standardized mean difference.

GWG was analyzed in 25 studies involving 4315 participants. A pooled analysis using a random-effects model showed that digital health interventions effectively controlled GWG compared with routine care (*I*²=54.2%; SMD –0.19, 95% CI –0.25 to –0.13; *P*<.001; Figure 3 [22-25,27-29,31,32,34,42-46,48,50,52,54-56,58,62,63]). Among these, 14 studies with 2675 participants reported the proportion of individuals exceeding the Institute of Medicine (IOM) recommendations for total weight gain during pregnancy, which are based on prepregnancy BMI categories for women: 12.5-18 kg for underweight (BMI<18.5 kg/m^2^), 11.5-16 kg for normal weight (BMI 18.5-24.9 kg/m^2^), 7-11.5 kg for overweight (BMI 25-29.9 kg/m^2^), and 5-9 kg for obesity (BMI≥30 kg/m^2^). In comparison, 10 studies with 1630 participants examined the proportion of women achieving sufficient weight gain according to these recommendations. Both analyses showed no heterogeneity (*I*²=0%, *P*=.45; *I*²=0%, *P=.*85, respectively).

**Figure 4:**
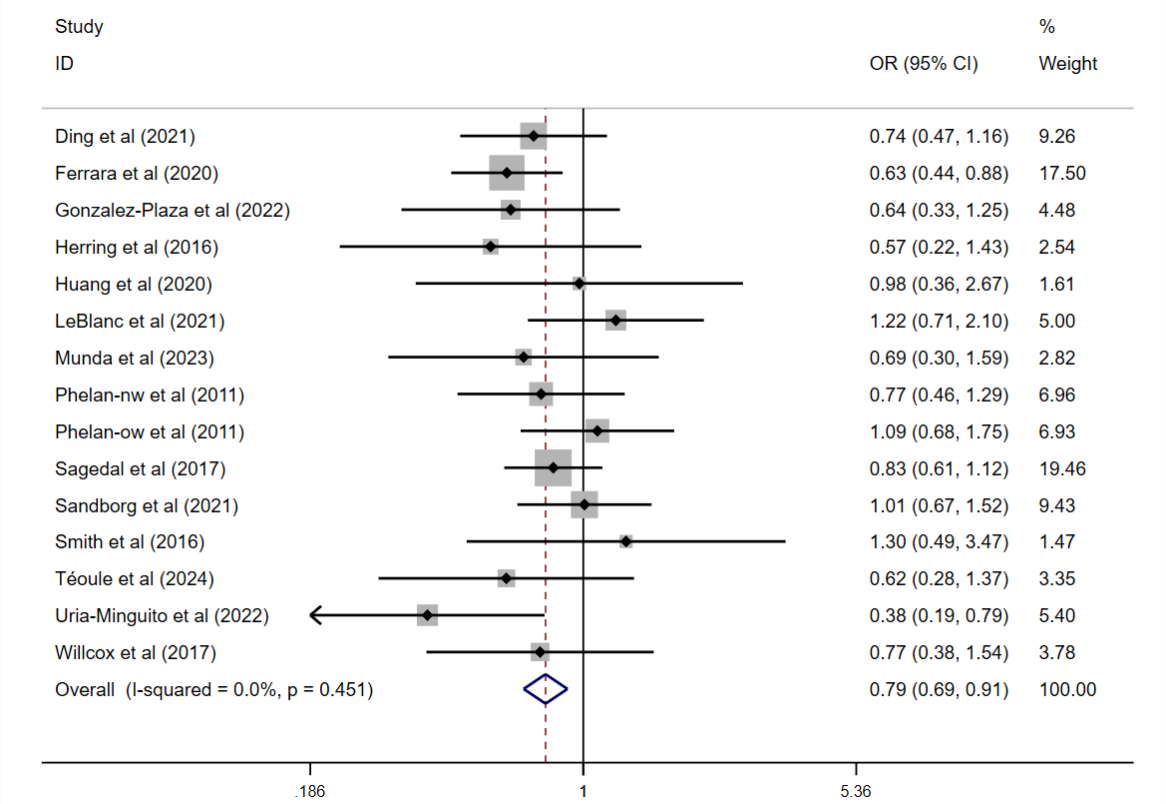
Effect on excessive Institute of Medicine (IOM) total weight gain. OR: odds ratio.

**Figure 5:**
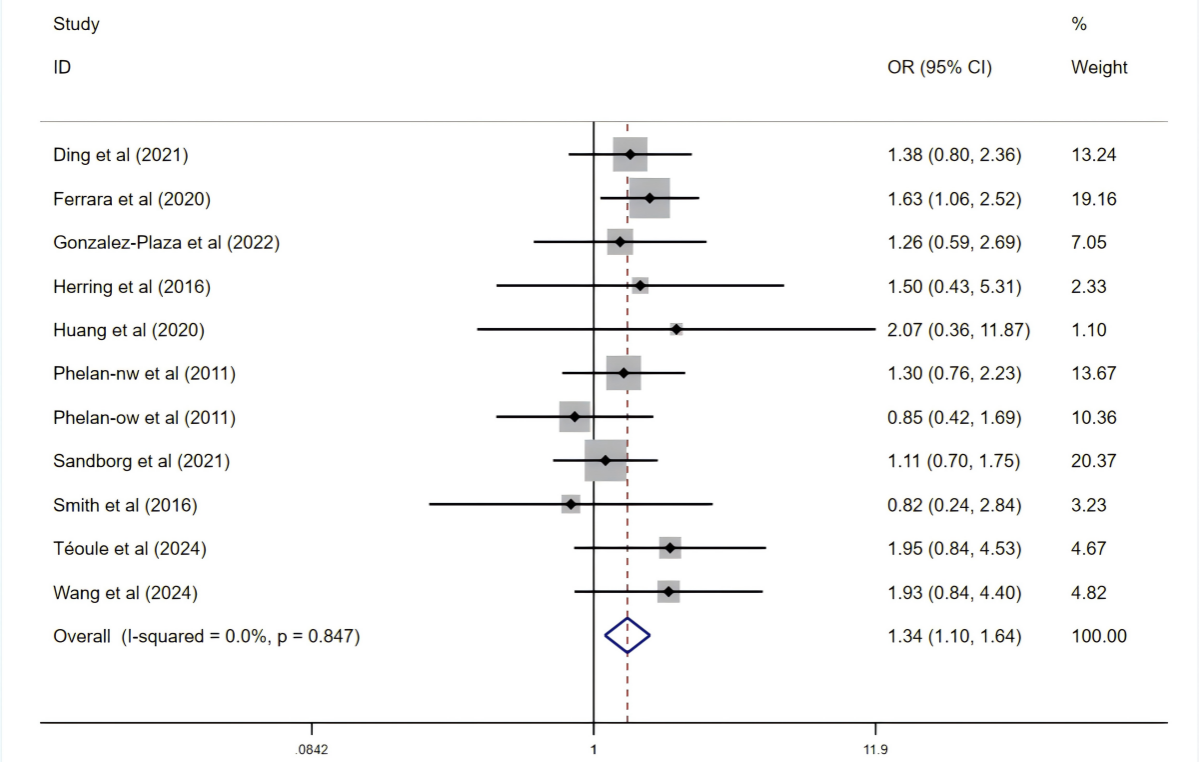
Effect on adequate Institute of Medicine (IOM) total weight gain. OR: odds ratio.

A fixed-effects model revealed a significant reduction in the proportion of women exceeding recommended GWG (OR 0.79, 95% CI 0.69-0.91; *P=.*001; Figure 4 [24,28,29,31,32,45,46,48-50,55,56,58,62]) and a significant increase in those meeting IOM GWG guidelines (OR 1.34, 95% CI 1.10-1.64; *P=.*003; Figure 5 [24,28,29,45,46,48,50,55,62,63]). However, no significant difference was observed in the proportion of participants falling below the IOM GWG guidelines.

Subgroup analysis of 6 studies revealed that overweight or obese participants experienced a weight gain reduction of 0.348 kg compared with the control group (*I*²=0%; SMD –0.35, 95% CI –0.45 to –0.24; *P* <.001). However, no significant difference was observed between participants with GDM and those without high-risk conditions.

#### Effect on Delivery Mode

A pooled analysis of 34 studies (n=147,382) found no statistically significant difference in cesarean section rates between the intervention and control groups (OR 1.03, 95% CI 0.99-1.06; *P*=.12), with no heterogeneity detected. Similarly, data from 13 studies (n=4450) showed no significant impact on vaginal delivery rates (OR 1.05, 95% CI 0.95-1.15; *P=.*37), with no evidence of heterogeneity in these findings.

#### Effect on Gestational Age

Analysis of gestational week at delivery across 23 studies (n=5330) revealed high heterogeneity (*I*²=94.8%; *P*<.001). Using a random-effects model, no statistically significant difference was observed between the intervention and control groups (SMD –0.004, 95% CI –0.27 to 0.26; *P*=.97).

#### Effect on Other Maternal Outcomes

**Figure 6:**
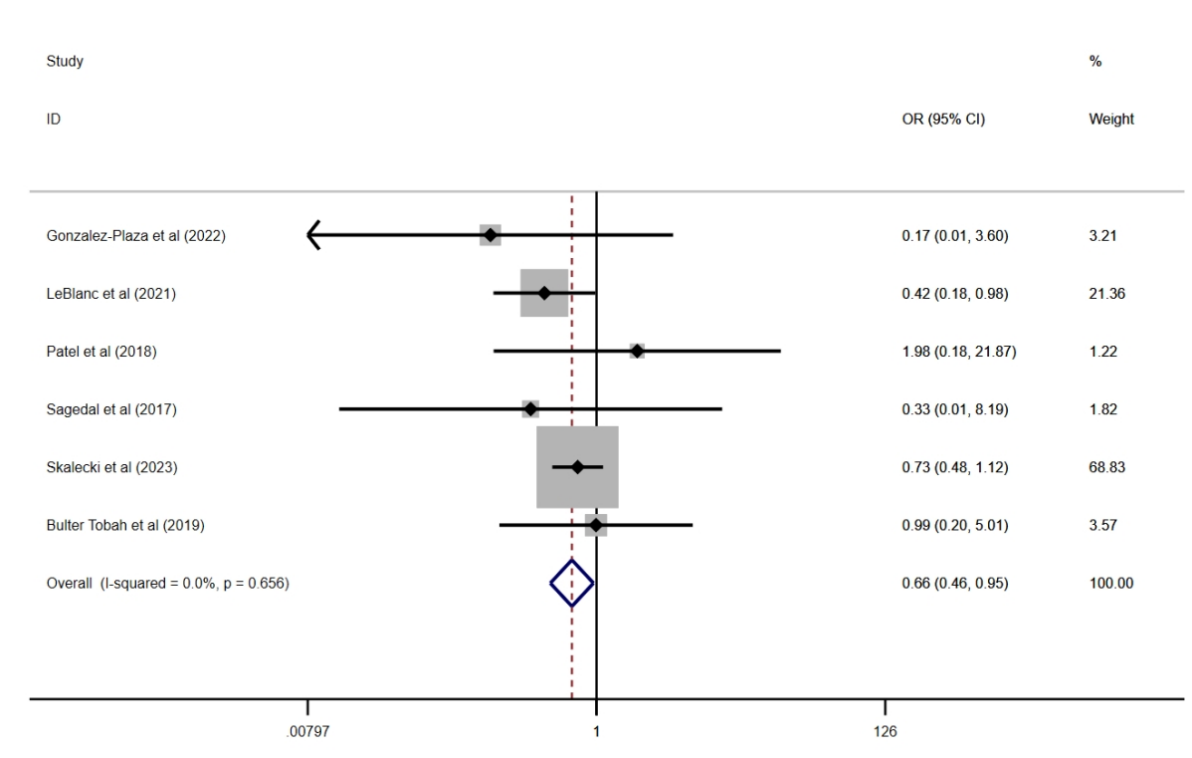
Effect on miscarriages. OR: odds ratio.

Miscarriage was reported in 6 studies involving 142,385 participants. These studies detected no heterogeneity (*I*²=0%; *P=.*66). A fixed-effects model revealed a statistically significant difference in miscarriage rates between the intervention and control groups (OR 0.66, 95% CI 0.46-0.95; *P=.*03; Figure 6 [32,37,44,49,55,60]). However, no significant differences were observed in the risk of shoulder dystocia, based on 4 studies (OR 0.35, 95% CI 0.12-1.02; *P*=.06), or in fasting blood glucose levels, analyzed in 11 studies (OR –0.16, 95% CI –0.32 to 0.01; *P*=.07).

A total of 12 studies (n=2769) assessed the prevalence of gestational diabetes, while 20 studies (n=3398) evaluated the incidence of gestational hypertension or preeclampsia. Both outcomes demonstrated low heterogeneity (*I*²=16.9%; *P*=.27 and *I*²=7.2%; *P*=.34, respectively). Using the Mantel-Haenszel fixed-effects model, no significant reduction was found in the prevalence of gestational diabetes (OR 0.87, 95% CI 0.70-1.08; *P=.*20) or gestational hypertension or preeclampsia (OR 0.88, 95% CI 0.70-1.11; *P=.*27).

#### Effects on Preterm Birth

**Figure 7:**
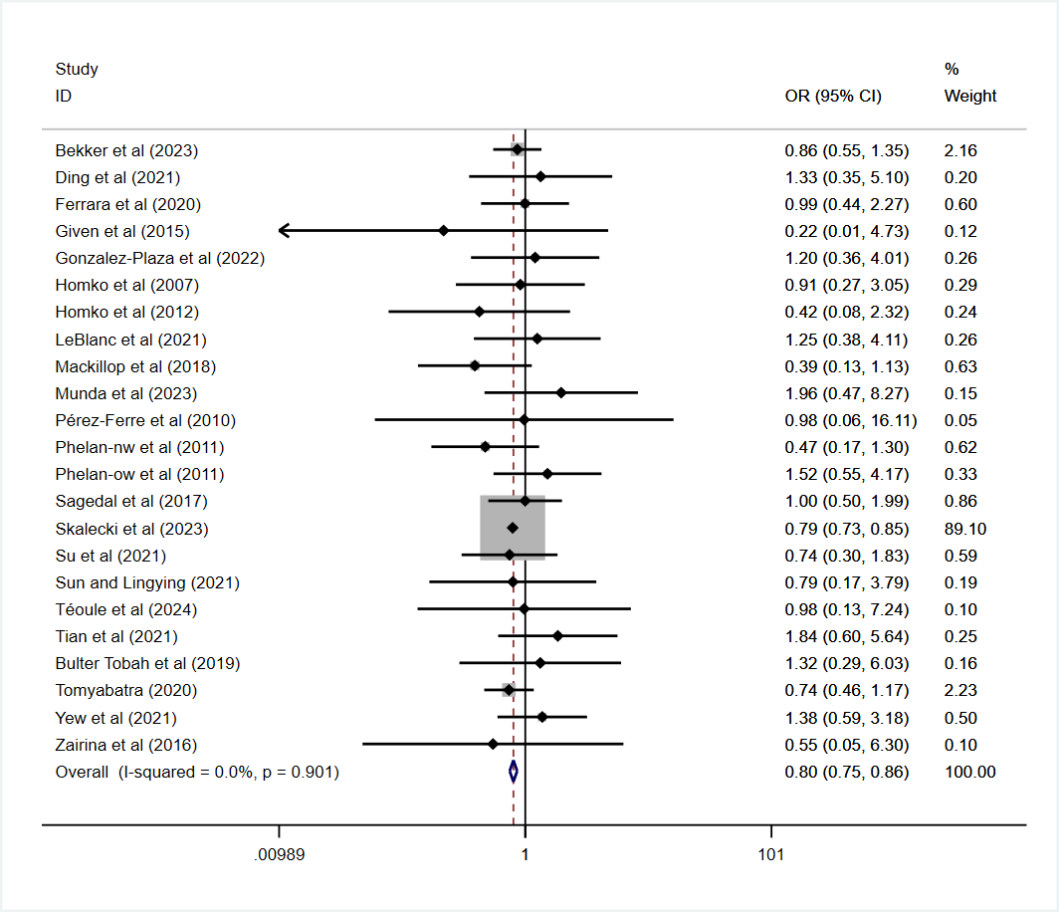
Effect on the prevalence of preterm birth. OR: odds ratio.

A pooled analysis of 22 studies involving 144,695 participants revealed a significantly lower prevalence of preterm births (before 37 weeks) among neonates in the intervention group compared to the control group (OR 0.80, 95% CI 0.75-0.86; *P*<.001; Figure 7 [22-26,30,32,35,44,45,47-49,51-55,57,58,60,62]). No heterogeneity was detected (*I*²=0%; *P*=.90).

#### Effects on Infant Circumstance

A total of 26 studies assessed neonatal birth weight (*I*²=39.6%; *P=.*02), and 6 examined birth length (*I*²=30.6%; *P*=.21). Compared with the control group, computer-based health interventions showed no statistically significant differences in birth weight (SMD 0.02, 95% CI –0.06 to 0.09, *P=.*71) or birth length (SMD –0.06, 95% CI –0.21 to 0.10; *P*=.48). Furthermore, 5 studies evaluated infant head circumference, but no significant difference was observed between the groups (SMD 0.02, 95% CI –0.10 to 0.14; *P*=.74).

#### Effects on SGA and LGA

**Figure 8:**
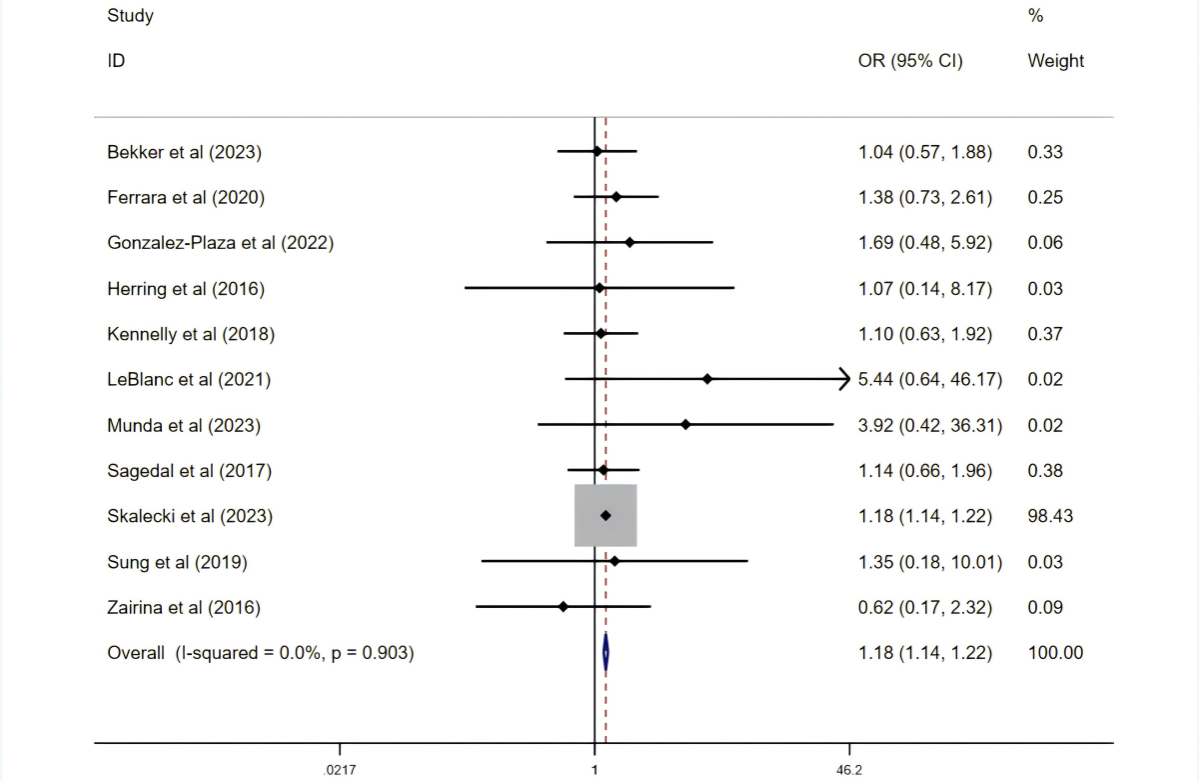
Effect on the prevalence of small for gestational age (SGA). OR: odds ratio.

A total of 11 RCTs involving 142,303 participants evaluated the incidence of SGA and showed no heterogeneity among the studies (*I*²=0%; *P=.*90). A fixed-effects model revealed a significant difference in SGA incidence between the intervention and control groups (OR 1.18, 95% CI 1.14-1.22; *P*<.001; Figure 8 [28,30,32,34,43,45,49,55,57,58,60]). In contrast, 13 studies (n=2403) assessed the incidence of LGA, also without heterogeneity, but the overall effect for LGA was not statistically significant (OR 0.91, 95% CI 0.69-1.19; *P*=.48).

#### Effects on Neonatal Complications

**Figure 9:**
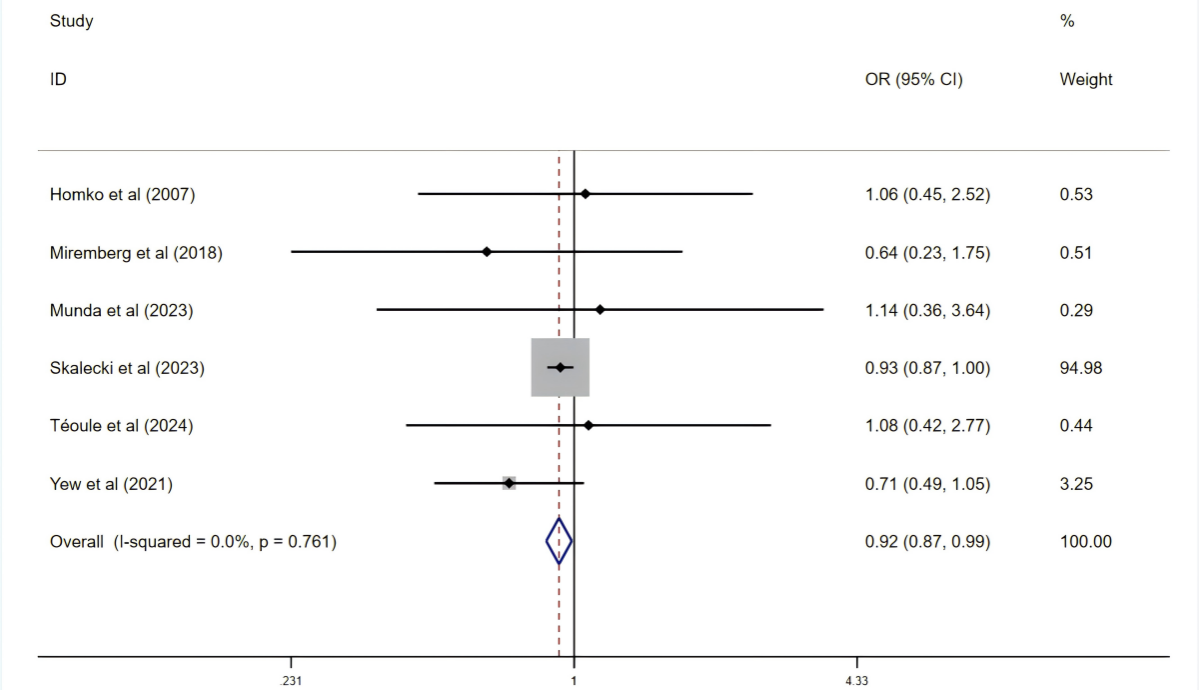
Effect on the prevalence of composite neonatal complications. OR: odds ratio.

In relation to neonatal complications, 12 studies addressed the issue of neonatal hypoglycemia (*I*²=0%; *P*=.94), 16 studies reviewed ICU admissions (*I*²=15.6%; *P*=.28), 8 studies mentioned jaundice or hyperbilirubinemia (*I*²=0%; *P=.*94), and 8 studies assessed respiratory distress syndrome (RDS; *I*²=4.6%; *P=.*39). For each condition, the intervention group exhibited no statistically significant decrease in incidence. However, pooled results from 6 studies involving 140,762 participants indicated that the digital health group experienced a significant decrease in combined complications (OR 0.93, 95% CI 0.87-0.98; *P=.*02; Figure 9 [22,36,54,58,60,62]), with no heterogeneity detected (*I*²=0%; *P*=.76).

#### Effects on Other Neonatal Outcomes

Based on 5 studies involving 481 newborns, the Apgar score analysis revealed no statistically significant difference between the intervention and control groups (SMD –0.11, 95% CI –0.29 to 0.07; *P*=.25). Similarly, 13 studies and 11 studies examined whether mHealth interventions reduced the risk of macrosomia (birth weight≥4000 g) and low birth weight (<2500 g), respectively, with no heterogeneity detected. Compared with routine care, the intervention group showed no significant improvement in the incidence of macrosomia (OR 0.90, 95% CI 0.72-1.14; *P=.*39) or low birth weight (OR 1.00; 95% CI 0.80-1.24; *P=.*97).

The results of the meta-analysis for 26 outcomes are summarized in Table 2.

**Table 2:** Meta-analysis results of 26 outcomes.

Outcomes	Studies, n	Participants, n			Heterogeneity				Statistical method		Effect estimate
		IG^a^	CG^b^	*I*^2^ (%)		*P* value					
GWG^c^	25	2155	2160	54.2		<.001		SMD^d^ (inverse variance, random, 95% CI)		–0.19 (–0.25 to –0.13)	
Proportion of exceeding IOM^e^ GWG	14	1340	1335	0		.45		OR^f^ (Mantel-Haenszel, fixed, 95% CI)		0.79 (0.69-0.91)	
Proportion of meeting IOM GWG	10	812	818	0		.85		OR (Mantel-Haenszel, fixed, 95% CI)		1.34 (1.10-1.64)	
Proportion of below IOM GWG	9	786	797	19.7		.26		OR (Mantel-Haenszel, fixed, 95% CI)		1.19 (0.91-1.55)	
Gestational diabetes	12	1380	1389	16.9		.27		OR (Mantel-Haenszel, fixed, 95% CI)		0.87 (0.70-1.08)	
Gestational hypertension or preeclampsia	20	1704	1694	7.2		.37		OR (Mantel-Haenszel, fixed, 95% CI)		0.88 (0.70-1.11)	
Miscarriage	6	14,947	127,438	0		.66		OR (Mantel-Haenszel, fixed, 95% CI)		0.66 (0.46-0.95)	
Cesarean delivery	34	17,467	129,915	0		.71		OR (Mantel-Haenszel, fixed, 95% CI)		1.03 (0.99-1.06)	
Vaginal delivery	14	2247	2203	0		.95		OR (Mantel-Haenszel, fixed, 95% CI)		1.05 (0.95-1.15)	
Shoulder dystocia	4	469	471	20.1		.29		OR (Mantel-Haenszel, fixed, 95% CI)		0.35 (0.12-1.02)	
Gestational age at delivery	23	2696	2634	94.8		<.001		SMD (inverse variance, random, 95% CI)		–0.004 (–0.27 to 0.26)	
Fasting blood glucose	11	788	773	57.4		.009		SMD (inverse variance, random, 95% CI)		–0.16 (–0.32 to 0.01)	
Preterm birth	22	16,115	128,580	0		.90		OR (Mantel-Haenszel, fixed, 95% CI)		0.80 (0.75-0.86 )	
Infant birth weight	26	2681	2618	39.6		.02		SMD (inverse variance, fixed, 95% CI)		0.02 (–0.06 to 0.09)	
Infant birth length	6	602	608	30.6		.21		SMD (inverse variance, fixed, 95% CI)		–0.06 (–0.21 to 0.10)	
Infant head circumference	5	563	563	0		.64		SMD (inverse variance, fixed, 95% CI)		0.02 (–0.10 to 0.14)	
Apgar score	5	241	240	0		.51		SMD (inverse variance, fixed, 95% CI)		–0.11 (–0.29 to 0.07)	
SGA^g^ (≤10%)	11	14,903	127,400	0		.90		OR (Mantel-Haenszel, fixed, 95% CI)		1.18 (1.14-1.22)	
LGA^h^ (≥90%)	13	1208	1195	0		.73		OR (Mantel-Haenszel, fixed, 95% CI)		0.91 (0.69-1.19)	
Macrosomia	17	1707	1692	0		.76		OR (Mantel-Haenszel, fixed, 95% CI)		0.90 (0.72-1.14)	
Birth weight ＜2500 g	10	2021	1965	0		.56		OR (Mantel-Haenszel, fixed, 95% CI)		1.00 (0.80-1.24)	
Neonatal hypoglycemia	12	784	755	0		.94		OR (Mantel-Haenszel, fixed, 95% CI)		0.87 (0.65-1.15)	
ICU^i^ admission	16	15,464	127,924	15.6		.28		OR (Mantel-Haenszel, fixed, 95% CI)		1.00 (0.93-1.08)	
Jaundice or hyperbilirubinemia	8	505	499	0		.94		OR (Mantel-Haenszel, fixed, 95% CI)		0.89 (0.61-1.30)	
RDS^j^	8	873	828	4.6		.39		OR (Mantel-Haenszel, fixed, 95% CI)		0.71 (0.49-1.03)	
Neonatal composite outcome	6	14,132	126,630	0		.76		OR (Mantel-Haenszel, fixed, 95% CI)		0.93 (0.87-0.99)	

^a^IG: intervention group.

^b^CG: control group

^c^GWG: gestational weight gain.

^d^SMD: standardized mean difference.

^e^IOM: Institute of Medicine.

^f^OR: odds ratio.

^g^SGA: small for gestational age.

^h^LGA: large for gestational age.

^i^ICU: intensive care unit.

^j^RDS: respiratory distress syndrome.

## Discussion

### Principal Findings

This meta-analysis revealed that digital health interventions significantly improved excessive GWG, reduced miscarriage and preterm birth incidence, and enhanced neonatal outcomes. However, the benefits for women with insufficient weight gain were limited, and an increased rate of infants who are SGA was observed.

### Comparison With Previous Work

This review evaluated the impact of digital health interventions on maternal and neonatal outcomes, with a particular focus on GWG. The findings revealed that digital interventions significantly improved GWG management among pregnant women. Compared to the control group, the intervention group showed a notable decrease in women exceeding recommended weight gain and increased adherence to the IOM GWG guidelines (*P*<.05). These results align with previous meta-analyses by Islam et al [64] and He et al [16]. However, the reduction in weight gain observed in our study (–0.145 kg) was less pronounced than the reductions reported by Islam et al [64] (–1.07 kg) and Antoun et al [65] (–1.99 kg). This discrepancy may be attributed to differences in the types and intensity of digital interventions and participant characteristics.

Subgroup analysis further revealed that significant GWG reductions were predominantly observed in participants who were overweight or obese rather than those with GDM. This may be explained by the intensive medical management typically provided to patients with GDM, which may diminish the additional benefits of digital interventions. These findings underscore the potential value of digital health care for individuals with prepregnancy overweight or obesity, as effective weight control in these populations is crucial. Excess weight not only increases the risk of pregnancy complications but also poses significant long-term health risks for their children [66].

The findings did not align with our hypothesis that digital health interventions would benefit participants experiencing insufficient GWG. This discrepancy may stem from the complex causes of inadequate weight gain during pregnancy, which include factors such as prepregnancy anemia, gestational diabetes, unhealthy lifestyle behaviors (eg, substance abuse), parity, and crowded living conditions [67]. Addressing these issues requires more than education on healthy diets, self-monitoring, or food supplementation alone. Additionally, 4 of the 9 studies on insufficient GWG focused on women with overweight and obese, with the digital health interventions targeting weight loss rather than promoting weight gain. This lack of individualized weight management strategies may have limited the effectiveness of the interventions in supporting appropriate weight gain for women below the recommended range.

In our study, while digital health interventions did not address insufficient GWG, they were associated with a reduction in miscarriage and preterm birth rates, as well as an increase in the prevalence of infants who are SGA. The reduction in miscarriage aligns with findings from Victa et al [68], which demonstrated that telemedicine monitoring during pregnancy significantly lowers miscarriage risk by enabling early detection of potential complications and timely medical intervention. In addition, the decreased risk of preterm birth and neonatal composite outcomes (*P*<.05) is consistent with the study by Guo et al [15], which highlighted the positive impact of digital health interventions on neonatal health.

Although insufficient GWG is known to increase the risk of miscarriage and preterm birth, our findings suggest that digital interventions may mitigate these risks through mechanisms beyond weight control. These interventions likely promote comprehensive health management by emphasizing nutritional intake, lifestyle improvements, and self-management efficacy, thereby improving maternal health and reducing risks associated with miscarriage and preterm labor, even without significantly addressing inadequate weight gain [69].

Further supporting this perspective, our analysis revealed a significant reduction in composite neonatal complications (*P*<.05), including conditions such as hypoglycemia, jaundice, and acute respiratory distress syndrome. By focusing on combined outcomes, the analysis provides a holistic view of the intervention’s benefits, which aligns more closely with real-world clinical scenarios and underscores the broader potential of digital health interventions to improve maternal and neonatal health.

Pooling data revealed an unexpected correlation between digital health interventions and a higher incidence of infants who are SGA. This outcome may be attributed to the characteristics of the study population, as 9 out of the 11 studies on SGA involved participants with high-risk factors—5 focused on overweight and obesity, 2 on GDM, and 2 on other conditions. Interventions targeting weight control likely resulted in overly strict weight gain or dietary restrictions, increasing the energy gap [70] and potentially impairing placental development [71]. In addition, including participants already at substantial risk for SGA-related conditions may have amplified the negative effects of these interventions. Moreover, the inclusion of participants already at substantial risk for SGA-related conditions may have amplified the observed effects, complicating the interpretation of the actual impact of digital health interventions on SGA. While subgroup analyses could provide more nuanced insights, the variability and limited availability of stratified BMI data across studies hindered such analyses. Future studies should prioritize detailed BMI stratification and reporting to better elucidate the differential impacts of digital health interventions on SGA incidence.

Despite this, digital health interventions offer significant benefits for women and newborns through multiple mechanisms. They provide evidence-based, tailored educational resources that address women’s specific needs [72,73] while encouraging active involvement in health monitoring. These tools enable women to track food intake, physical activity, and physical parameters such as weight changes and blood pressure [74], with positive feedback reinforcing their ability to manage their health effectively. Moreover, some intervention platforms facilitate online communication with skilled clinical and nursing personnel, reducing pregnancy-related stress and enhancing health literacy [75]. These multifaceted approaches contribute to improved maternal and neonatal outcomes, even when specific challenges persist.

### Strengths and Limitations

The strengths of this study encompass its inclusive population, as it considered all pregnant women rather than concentrating exclusively on specific subgroups such as those with diabetes or obesity, thereby enhancing the generalizability of the findings. In addition, we used a wide range of outcome measures, providing a comprehensive understanding of the impact of digital health interventions on maternal and neonatal health. Furthermore, our study incorporated the most recent literature, ensuring relevance to current clinical practice.

However, the study also has limitations. First, we expanded the search terms to include a broader range of eligible studies, thus enabling a more comprehensive investigation. Nevertheless, the quantity of published RCTs was restricted, indicating that the findings of our study necessitate careful interpretation and require further validation through supplementary research. Second, the follow-up period for the majority of the included studies concluded at birth, indicating a need for further investigation into the long-term effects on women and children to understand the implications of our findings fully. Third, the large sample size in the study by Skalecki et al [60], which focused on telemonitoring fetal movement, may influence the overall results. While a larger sample size is often beneficial for increasing the statistical power of a study, it may also introduce a bias toward the generalizability of the findings, especially in the context of the intervention types and outcome measures. A more specific analysis of how large sample sizes in studies with different intervention types could affect the overall results and the generalizability of the findings would provide a more comprehensive perspective. Fourth, the included studies encompassed a heterogeneous population of pregnant women with varying BMI categories (eg, underweight, normal weight, overweight, and obesity). However, some studies did not stratify participants by prepregnancy BMI or provide detailed subgroup analyses. This lack of consistent BMI reporting limits our ability to interpret the differential effects of digital health interventions across BMI subgroups. Future studies with standardized BMI stratification and detailed subgroup reporting are essential to delineate these effects better. Fifth, while the reduction in GWG observed in our study has general clinical significance due to the known risks of excessive weight gain, the lack of specific data on other contextual factors, such as dietary habits, physical activity levels, and socioeconomic status, may affect the interpretation of the results. These factors could mediate the relationship between digital health interventions and GWG, highlighting the need for more comprehensive datasets in future research.

### Further Research

Further studies should investigate the potential differential impacts of these interventions across various populations, considering factors such as age, ethnicity, and health conditions, and determine the most effective digital intervention tailored to participants’ backgrounds.

### Conclusion

Digital health interventions facilitate maternal weight gain, which later influences neonatal health outcomes by decreasing complications such as miscarriages, preterm birth, and combined neonatal complications.

## Data Availability

The datasets generated or analyzed during this study are available from the corresponding author on reasonable request.

## Appendix

Multimedia Appendix 1 PRISMA (Preferred Reporting Items for Systematic Reviews and Meta-Analyses) checklist.

Multimedia Appendix 2 Details of the search.

Multimedia Appendix 3 Publication bias.

## Abbreviations

GDM: gestational diabetes mellitus
GWG: gestational weight gain
IOM: Institute of Medicine
LGA: large for gestational age
mHealth: mobile health
OR: odds ratio
PRISMA: Preferred Reporting Items for Systematic Reviews and Meta-Analyses
RCT: randomized controlled trial
RoB 2: Revised Cochrane risk-of-bias tool for randomized trials
SGA: small for gestational age
SMD: standardized mean difference
