# Supplementary material for: Association of Digital Health Interventions With Maternal and Neonatal Outcomes: Systematic Review and Meta-Analysis
Source: J Med Internet Res. 2025 Mar 14;27:e66580. doi: 10.2196/66580 (PMC11953608; doi:10.2196/66580)
Supplement: Multimedia Appendix 2 [file jmir_v27i1e66580_app2.doc]

**Multimedia Appendix 2.** Search terms in 4 databases and results

**1.**Searching results of Pubmed library：

| **Search number** | **Query** | **Sort By** | **Filters** | **Search Details** | **Results** | **Time** |
| --- | --- | --- | --- | --- | --- | --- |
| 1 | ((((((((((((Phone[Title/Abstract]) OR (Mobile phone[Title/Abstract])) OR (cell phone[Title/Abstract])) OR (cellular phone[Title/Abstract])) OR (smart phone[Title/Abstract])) OR (telephone[Title/Abstract])) OR (iphone[Title/Abstract])) OR (Ipad[Title/Abstract])) OR (Ipod[Title/Abstract])) OR (iso[Title/Abstract])) OR (android[Title/Abstract])) OR (Computers[Title/Abstract])) OR (tablet[Title/Abstract]) |  |  | "Phone"[Title/Abstract] OR "mobile phone"[Title/Abstract] OR "cell phone"[Title/Abstract] OR "cellular phone"[Title/Abstract] OR "smart phone"[Title/Abstract] OR "telephone"[Title/Abstract] OR "iphone"[Title/Abstract] OR "Ipad"[Title/Abstract] OR "Ipod"[Title/Abstract] OR "iso"[Title/Abstract] OR "android"[Title/Abstract] OR "Computers"[Title/Abstract] OR "tablet"[Title/Abstract] | 206,962 | 2:30:36 |
| 2 | "intervention"[Title/Abstract] |  |  | "intervention"[Title/Abstract] | 878,013 | 2:31:50 |
| 3 | #1 AND #2 |  |  | ("Phone"[Title/Abstract] OR "mobile phone"[Title/Abstract] OR "cell phone"[Title/Abstract] OR "cellular phone"[Title/Abstract] OR "smart phone"[Title/Abstract] OR "telephone"[Title/Abstract] OR "iphone"[Title/Abstract] OR "Ipad"[Title/Abstract] OR "Ipod"[Title/Abstract] OR "iso"[Title/Abstract] OR "android"[Title/Abstract] OR "Computers"[Title/Abstract] OR "tablet"[Title/Abstract]) AND "intervention"[Title/Abstract] | 23,586 | 2:32:07 |
| 4 | "application"[Title/Abstract] OR "app"[Title/Abstract] |  |  | "application"[Title/Abstract] OR "app"[Title/Abstract] | 1,160,392 | 2:32:22 |
| 5 | #1 AND #4 |  |  | ("Phone"[Title/Abstract] OR "mobile phone"[Title/Abstract] OR "cell phone"[Title/Abstract] OR "cellular phone"[Title/Abstract] OR "smart phone"[Title/Abstract] OR "telephone"[Title/Abstract] OR "iphone"[Title/Abstract] OR "Ipad"[Title/Abstract] OR "Ipod"[Title/Abstract] OR "iso"[Title/Abstract] OR "android"[Title/Abstract] OR "Computers"[Title/Abstract] OR "tablet"[Title/Abstract]) AND ("application"[Title/Abstract] OR "app"[Title/Abstract]) | 16,332 | 2:32:43 |
| 6 | #3 OR #5 |  |  | (("Phone"[Title/Abstract] OR "mobile phone"[Title/Abstract] OR "cell phone"[Title/Abstract] OR "cellular phone"[Title/Abstract] OR "smart phone"[Title/Abstract] OR "telephone"[Title/Abstract] OR "iphone"[Title/Abstract] OR "Ipad"[Title/Abstract] OR "Ipod"[Title/Abstract] OR "iso"[Title/Abstract] OR "android"[Title/Abstract] OR "Computers"[Title/Abstract] OR "tablet"[Title/Abstract]) AND "intervention"[Title/Abstract]) OR (("Phone"[Title/Abstract] OR "mobile phone"[Title/Abstract] OR "cell phone"[Title/Abstract] OR "cellular phone"[Title/Abstract] OR "smart phone"[Title/Abstract] OR "telephone"[Title/Abstract] OR "iphone"[Title/Abstract] OR "Ipad"[Title/Abstract] OR "Ipod"[Title/Abstract] OR "iso"[Title/Abstract] OR "android"[Title/Abstract] OR "Computers"[Title/Abstract] OR "tablet"[Title/Abstract]) AND ("application"[Title/Abstract] OR "app"[Title/Abstract])) | 37,134 | 2:33:09 |
| 7 | (((Wearable device[Title/Abstract]) OR (wearable technology[Title/Abstract])) OR (portable device[Title/Abstract])) OR (mobile device[Title/Abstract]) |  |  | "wearable device"[Title/Abstract] OR "wearable technology"[Title/Abstract] OR "portable device"[Title/Abstract] OR "mobile device"[Title/Abstract] | 8,042 | 2:33:26 |
| 8 | (((((text message[Title/Abstract]) OR (multimedia[Title/Abstract])) OR (multi‐media[Title/Abstract])) OR (SMS[Title/Abstract])) OR (web-based[Title/Abstract])) OR (internet based[Title/Abstract]) |  |  | "text message"[Title/Abstract] OR "multimedia"[Title/Abstract] OR "multi-media"[Title/Abstract] OR "SMS"[Title/Abstract] OR "web-based"[Title/Abstract] OR "internet based"[Title/Abstract] | 75,541 | 2:33:45 |
| 9 | ((mobile health[Title/Abstract]) OR (mobile health technology[Title/Abstract])) OR (mobile tech[Title/Abstract]) |  |  | "mobile health"[Title/Abstract] OR "mobile health technology"[Title/Abstract] OR "mobile tech"[Title/Abstract] | 10,138 | 2:34:05 |
| 10 | ((((online[Title/Abstract]) OR (online communication[Title/Abstract])) OR (on-line communication[Title/Abstract])) OR (online intervention[Title/Abstract])) OR (telecommunication[Title/Abstract]) |  |  | "online"[Title/Abstract] OR "online communication"[Title/Abstract] OR "on line communication"[Title/Abstract] OR "online intervention"[Title/Abstract] OR "telecommunication"[Title/Abstract] | 277,748 | 2:34:20 |
| 11 | (((e-mail based[Title/Abstract]) OR (email-based[Title/Abstract])) OR (email based[Title/Abstract])) OR (electronic mail based[Title/Abstract]) |  |  | "e mail based"[Title/Abstract] OR "email-based"[Title/Abstract] OR "email-based"[Title/Abstract] OR (("electronical"[All Fields] OR "electronically"[All Fields] OR "electronics"[MeSH Terms] OR "electronics"[All Fields] OR "electronic"[All Fields]) AND "mail based"[Title/Abstract]) | 376 | 2:34:45 |
| 12 | (((((((((((((((((((((e-health[Title/Abstract]) OR (ehealth[Title/Abstract])) OR (eHealth[Title/Abstract])) OR (electronic health[Title/Abstract])) OR (eTherap*[Title/Abstract])) OR (e-Therap*[Title/Abstract])) OR (tele-health[Title/Abstract])) OR (telehealth[Title/Abstract])) OR (tele-care[Title/Abstract])) OR (telecare[Title/Abstract])) OR (telehealthcare[Title/Abstract])) OR (tele-healthcare[Title/Abstract])) OR (tele-homecare[Title/Abstract])) OR (telemedicine[Title/Abstract])) OR (tele-medicine[Title/Abstract])) OR (telemental health[Title/Abstract])) OR (Telerehabilitation[Title/Abstract])) OR (tele-rehabilitation[Title/Abstract])) OR (telepsychiatry[Title/Abstract])) OR (teletherap*[Title/Abstract])) OR (Therapy, Computer-Assisted[Title/Abstract])) OR (Connected Health[Title/Abstract]) |  |  | "e-health"[Title/Abstract] OR "eHealth"[Title/Abstract] OR "eHealth"[Title/Abstract] OR "electronic health"[Title/Abstract] OR "etherap*"[Title/Abstract] OR "e therap*"[Title/Abstract] OR "tele-health"[Title/Abstract] OR "telehealth"[Title/Abstract] OR "tele-care"[Title/Abstract] OR "telecare"[Title/Abstract] OR "telehealthcare"[Title/Abstract] OR "tele-healthcare"[Title/Abstract] OR "tele-homecare"[Title/Abstract] OR "telemedicine"[Title/Abstract] OR "tele-medicine"[Title/Abstract] OR "telemental health"[Title/Abstract] OR "Telerehabilitation"[Title/Abstract] OR "tele-rehabilitation"[Title/Abstract] OR "telepsychiatry"[Title/Abstract] OR "teletherap*"[Title/Abstract] OR "therapy computer assisted"[Title/Abstract] OR "connected health"[Title/Abstract] | 91,339 | 2:35:03 |
| 13 | ((((((telemetry[Title/Abstract]) OR (telemonitor[Title/Abstract])) OR (telemonitoring[Title/Abstract])) OR (tele-monitoring[Title/Abstract])) OR (Remote Sensing Technology[Title/Abstract])) OR (smartphone technology[Title/Abstract])) OR (virtual reality[Title/Abstract]) |  |  | "telemetry"[Title/Abstract] OR "telemonitor"[Title/Abstract] OR "telemonitoring"[Title/Abstract] OR "tele-monitoring"[Title/Abstract] OR "remote sensing technology"[Title/Abstract] OR "smartphone technology"[Title/Abstract] OR "virtual reality"[Title/Abstract] | 33,056 | 2:36:43 |
| 14 | ((((facebook[Title/Abstract]) OR (wechat[Title/Abstract])) OR (twitter[Title/Abstract])) OR (microblog[Title/Abstract])) OR (weibo[Title/Abstract]) |  |  | "facebook"[Title/Abstract] OR "wechat"[Title/Abstract] OR "twitter"[Title/Abstract] OR "microblog"[Title/Abstract] OR "weibo"[Title/Abstract] | 15,064 | 2:36:59 |
| 15 | ((digital health[Title/Abstract]) OR (digital medicine system[Title/Abstract])) OR (digital behavior change[Title/Abstract]) |  |  | "digital health"[Title/Abstract] OR "digital medicine system"[Title/Abstract] OR "digital behavior change"[Title/Abstract] | 10,646 | 2:37:14 |
| 16 | (((e-care[Title/Abstract]) OR (e-consultation[Title/Abstract])) OR (E-counselling[Title/Abstract])) OR (remote consultation[Title/Abstract]) |  |  | "e-care"[Title/Abstract] OR "e-consultation"[Title/Abstract] OR "E-counselling"[Title/Abstract] OR "remote consultation"[Title/Abstract] | 1,151 | 2:37:28 |
| 17 | #6 OR #7 OR #8 OR #9 OR #10 OR #11 OR #12 OR #13 OR #14 OR #15 OR #16 |  |  | (("Phone"[Title/Abstract] OR "mobile phone"[Title/Abstract] OR "cell phone"[Title/Abstract] OR "cellular phone"[Title/Abstract] OR "smart phone"[Title/Abstract] OR "telephone"[Title/Abstract] OR "iphone"[Title/Abstract] OR "Ipad"[Title/Abstract] OR "Ipod"[Title/Abstract] OR "iso"[Title/Abstract] OR "android"[Title/Abstract] OR "Computers"[Title/Abstract] OR "tablet"[Title/Abstract]) AND "intervention"[Title/Abstract]) OR (("Phone"[Title/Abstract] OR "mobile phone"[Title/Abstract] OR "cell phone"[Title/Abstract] OR "cellular phone"[Title/Abstract] OR "smart phone"[Title/Abstract] OR "telephone"[Title/Abstract] OR "iphone"[Title/Abstract] OR "Ipad"[Title/Abstract] OR "Ipod"[Title/Abstract] OR "iso"[Title/Abstract] OR "android"[Title/Abstract] OR "Computers"[Title/Abstract] OR "tablet"[Title/Abstract]) AND ("application"[Title/Abstract] OR "app"[Title/Abstract])) OR ("wearable device"[Title/Abstract] OR "wearable technology"[Title/Abstract] OR "portable device"[Title/Abstract] OR "mobile device"[Title/Abstract]) OR ("text message"[Title/Abstract] OR "multimedia"[Title/Abstract] OR "multi-media"[Title/Abstract] OR "SMS"[Title/Abstract] OR "web-based"[Title/Abstract] OR "internet based"[Title/Abstract]) OR ("mobile health"[Title/Abstract] OR "mobile health technology"[Title/Abstract] OR "mobile tech"[Title/Abstract]) OR ("online"[Title/Abstract] OR "online communication"[Title/Abstract] OR "on line communication"[Title/Abstract] OR "online intervention"[Title/Abstract] OR "telecommunication"[Title/Abstract]) OR ("e mail based"[Title/Abstract] OR "email-based"[Title/Abstract] OR "email-based"[Title/Abstract] OR (("electronical"[All Fields] OR "electronically"[All Fields] OR "electronics"[MeSH Terms] OR "electronics"[All Fields] OR "electronic"[All Fields]) AND "mail based"[Title/Abstract])) OR ("e-health"[Title/Abstract] OR "eHealth"[Title/Abstract] OR "eHealth"[Title/Abstract] OR "electronic health"[Title/Abstract] OR "etherap*"[Title/Abstract] OR "e therap*"[Title/Abstract] OR "tele-health"[Title/Abstract] OR "telehealth"[Title/Abstract] OR "tele-care"[Title/Abstract] OR "telecare"[Title/Abstract] OR "telehealthcare"[Title/Abstract] OR "tele-healthcare"[Title/Abstract] OR "tele-homecare"[Title/Abstract] OR "telemedicine"[Title/Abstract] OR "tele-medicine"[Title/Abstract] OR "telemental health"[Title/Abstract] OR "Telerehabilitation"[Title/Abstract] OR "tele-rehabilitation"[Title/Abstract] OR "telepsychiatry"[Title/Abstract] OR "teletherap*"[Title/Abstract] OR "therapy computer assisted"[Title/Abstract] OR "connected health"[Title/Abstract]) OR ("telemetry"[Title/Abstract] OR "telemonitor"[Title/Abstract] OR "telemonitoring"[Title/Abstract] OR "tele-monitoring"[Title/Abstract] OR "remote sensing technology"[Title/Abstract] OR "smartphone technology"[Title/Abstract] OR "virtual reality"[Title/Abstract]) OR ("facebook"[Title/Abstract] OR "wechat"[Title/Abstract] OR "twitter"[Title/Abstract] OR "microblog"[Title/Abstract] OR "weibo"[Title/Abstract]) OR ("digital health"[Title/Abstract] OR "digital medicine system"[Title/Abstract] OR "digital behavior change"[Title/Abstract]) OR ("e-care"[Title/Abstract] OR "e-consultation"[Title/Abstract] OR "E-counselling"[Title/Abstract] OR "remote consultation"[Title/Abstract]) | 507,661 | 2:37:41 |
| 18 | pregnancy[MeSH Terms] |  |  | "pregnancy"[MeSH Terms] | 1,036,715 | 2:38:01 |
| 19 | #17 AND #18 |  |  | ((("Phone"[Title/Abstract] OR "mobile phone"[Title/Abstract] OR "cell phone"[Title/Abstract] OR "cellular phone"[Title/Abstract] OR "smart phone"[Title/Abstract] OR "telephone"[Title/Abstract] OR "iphone"[Title/Abstract] OR "Ipad"[Title/Abstract] OR "Ipod"[Title/Abstract] OR "iso"[Title/Abstract] OR "android"[Title/Abstract] OR "Computers"[Title/Abstract] OR "tablet"[Title/Abstract]) AND "intervention"[Title/Abstract]) OR (("Phone"[Title/Abstract] OR "mobile phone"[Title/Abstract] OR "cell phone"[Title/Abstract] OR "cellular phone"[Title/Abstract] OR "smart phone"[Title/Abstract] OR "telephone"[Title/Abstract] OR "iphone"[Title/Abstract] OR "Ipad"[Title/Abstract] OR "Ipod"[Title/Abstract] OR "iso"[Title/Abstract] OR "android"[Title/Abstract] OR "Computers"[Title/Abstract] OR "tablet"[Title/Abstract]) AND ("application"[Title/Abstract] OR "app"[Title/Abstract])) OR ("wearable device"[Title/Abstract] OR "wearable technology"[Title/Abstract] OR "portable device"[Title/Abstract] OR "mobile device"[Title/Abstract]) OR ("text message"[Title/Abstract] OR "multimedia"[Title/Abstract] OR "multi-media"[Title/Abstract] OR "SMS"[Title/Abstract] OR "web-based"[Title/Abstract] OR "internet based"[Title/Abstract]) OR ("mobile health"[Title/Abstract] OR "mobile health technology"[Title/Abstract] OR "mobile tech"[Title/Abstract]) OR ("online"[Title/Abstract] OR "online communication"[Title/Abstract] OR "on line communication"[Title/Abstract] OR "online intervention"[Title/Abstract] OR "telecommunication"[Title/Abstract]) OR ("e mail based"[Title/Abstract] OR "email-based"[Title/Abstract] OR "email-based"[Title/Abstract] OR (("electronical"[All Fields] OR "electronically"[All Fields] OR "electronics"[MeSH Terms] OR "electronics"[All Fields] OR "electronic"[All Fields]) AND "mail based"[Title/Abstract])) OR ("e-health"[Title/Abstract] OR "eHealth"[Title/Abstract] OR "eHealth"[Title/Abstract] OR "electronic health"[Title/Abstract] OR "etherap*"[Title/Abstract] OR "e therap*"[Title/Abstract] OR "tele-health"[Title/Abstract] OR "telehealth"[Title/Abstract] OR "tele-care"[Title/Abstract] OR "telecare"[Title/Abstract] OR "telehealthcare"[Title/Abstract] OR "tele-healthcare"[Title/Abstract] OR "tele-homecare"[Title/Abstract] OR "telemedicine"[Title/Abstract] OR "tele-medicine"[Title/Abstract] OR "telemental health"[Title/Abstract] OR "Telerehabilitation"[Title/Abstract] OR"tele-rehabilitation"[Title/Abstract] OR "telepsychiatry"[Title/Abstract] OR "teletherap*"[Title/Abstract] OR "therapy computer assisted"[Title/Abstract] OR "connected health"[Title/Abstract]) OR ("telemetry"[Title/Abstract] OR "telemonitor"[Title/Abstract] OR "telemonitoring"[Title/Abstract] OR "tele-monitoring"[Title/Abstract] OR "remote sensing technology"[Title/Abstract] OR "smartphone technology"[Title/Abstract] OR "virtual reality"[Title/Abstract]) OR ("facebook"[Title/Abstract] OR "wechat"[Title/Abstract] OR "twitter"[Title/Abstract] OR "microblog"[Title/Abstract] OR "weibo"[Title/Abstract]) OR ("digital health"[Title/Abstract] OR "digital medicine system"[Title/Abstract] OR "digital behavior change"[Title/Abstract]) OR ("e-care"[Title/Abstract] OR "e-consultation"[Title/Abstract] OR "E-counselling"[Title/Abstract] OR "remote consultation"[Title/Abstract])) AND "pregnancy"[MeSH Terms] | 10,228 | 2:48:32 |
| 20 | #17 AND #18 |  | Randomized Controlled Trial | (((("Phone"[Title/Abstract] OR "mobile phone"[Title/Abstract] OR "cell phone"[Title/Abstract] OR "cellular phone"[Title/Abstract] OR "smart phone"[Title/Abstract] OR "telephone"[Title/Abstract] OR "iphone"[Title/Abstract] OR "Ipad"[Title/Abstract] OR "Ipod"[Title/Abstract] OR "iso"[Title/Abstract] OR "android"[Title/Abstract] OR "Computers"[Title/Abstract] OR "tablet"[Title/Abstract]) AND "intervention"[Title/Abstract]) OR (("Phone"[Title/Abstract] OR "mobile phone"[Title/Abstract] OR "cell phone"[Title/Abstract] OR "cellular phone"[Title/Abstract] OR "smart phone"[Title/Abstract] OR "telephone"[Title/Abstract] OR "iphone"[Title/Abstract] OR "Ipad"[Title/Abstract] OR "Ipod"[Title/Abstract] OR "iso"[Title/Abstract] OR "android"[Title/Abstract] OR "Computers"[Title/Abstract] OR "tablet"[Title/Abstract]) AND ("application"[Title/Abstract] OR "app"[Title/Abstract])) OR ("wearable device"[Title/Abstract] OR "wearable technology"[Title/Abstract] OR "portable device"[Title/Abstract] OR "mobile device"[Title/Abstract]) OR ("text message"[Title/Abstract] OR "multimedia"[Title/Abstract] OR "multi-media"[Title/Abstract] OR "SMS"[Title/Abstract] OR "web-based"[Title/Abstract] OR "internet based"[Title/Abstract]) OR ("mobile health"[Title/Abstract] OR "mobile health technology"[Title/Abstract] OR "mobile tech"[Title/Abstract]) OR ("online"[Title/Abstract] OR "online communication"[Title/Abstract] OR "on line communication"[Title/Abstract] OR "online intervention"[Title/Abstract] OR "telecommunication"[Title/Abstract]) OR ("e mail based"[Title/Abstract] OR "email-based"[Title/Abstract] OR "email-based"[Title/Abstract] OR (("electronical"[All Fields] OR "electronically"[All Fields] OR "electronics"[MeSH Terms] OR "electronics"[All Fields] OR "electronic"[All Fields]) AND "mail based"[Title/Abstract])) OR ("e-health"[Title/Abstract] OR "eHealth"[Title/Abstract] OR "eHealth"[Title/Abstract] OR "electronic health"[Title/Abstract] OR "etherap*"[Title/Abstract] OR "e therap*"[Title/Abstract] OR "tele-health"[Title/Abstract] OR "telehealth"[Title/Abstract] OR "tele-care"[Title/Abstract] OR "telecare"[Title/Abstract] OR "telehealthcare"[Title/Abstract] OR "tele-healthcare"[Title/Abstract] OR "tele-homecare"[Title/Abstract] OR "telemedicine"[Title/Abstract] OR "tele-medicine"[Title/Abstract] OR "telemental health"[Title/Abstract] OR "Telerehabilitation"[Title/Abstract] OR "tele-rehabilitation"[Title/Abstract] OR "telepsychiatry"[Title/Abstract] OR "teletherap*"[Title/Abstract] OR "therapy computer assisted"[Title/Abstract] OR "connected health"[Title/Abstract]) OR ("telemetry"[Title/Abstract] OR "telemonitor"[Title/Abstract] OR "telemonitoring"[Title/Abstract] OR "tele-monitoring"[Title/Abstract] OR "remote sensing technology"[Title/Abstract] OR "smartphone technology"[Title/Abstract] OR "virtual reality"[Title/Abstract]) OR ("facebook"[Title/Abstract] OR "wechat"[Title/Abstract] OR "twitter"[Title/Abstract] OR "microblog"[Title/Abstract] OR "weibo"[Title/Abstract]) OR ("digital health"[Title/Abstract] OR "digital medicine system"[Title/Abstract] OR "digital behavior change"[Title/Abstract]) OR ("e-care"[Title/Abstract] OR "e-consultation"[Title/Abstract] OR "E-counselling"[Title/Abstract] OR "remote consultation"[Title/Abstract])) AND "pregnancy"[MeSH Terms]) AND (randomizedcontrolledtrial[Filter]) | 973 | 2:48:48 |

**2.Searching results of Embase database**

| No. | Query | Results | Date |
| --- | --- | --- | --- |
| #1 | 'mobile phone':ab,ti OR 'phone':ab,ti OR 'cell phone':ab,ti OR 'cellular phone':ab,ti OR 'smart phone':ab,ti OR 'telephone':ab,ti OR 'iphone':ab,ti OR 'ipad':ab,ti OR 'ipod':ab,ti OR 'iso':ab,ti OR 'android':ab,ti OR 'computers':ab,ti OR 'tablet':ab,ti | 298564 | 21-Aug-24 |
| #2 | 'intervention':ab,ti | 1216186 | 21-Aug-24 |
| #3 | #1 AND #2 | 33054 | 21-Aug-24 |
| #4 | 'application':ab,ti OR 'app':ab,ti | 1394914 | 21-Aug-24 |
| #5 | #1 AND #4 | 22411 | 21-Aug-24 |
| #6 | #3 OR #5 | 52568 | 21-Aug-24 |
| #7 | 'wearable device':ab,ti OR 'wearable technology':ab,ti OR 'portable device':ab,ti OR 'mobile device':ab,ti | 9161 | 21-Aug-24 |
| #8 | 'mobile health':ab,ti OR 'mobile health technology':ab,ti OR 'mobile tech':ab,ti | 7775 | 21-Aug-24 |
| #9 | 'text message':ab,ti OR 'multimedia':ab,ti OR 'multi-media':ab,ti OR 'sms':ab,ti OR 'web-based':ab,ti OR 'internet based':ab,ti | 100019 | 21-Aug-24 |
| #10 | 'online':ab,ti OR 'online communication':ab,ti OR 'on-line communication':ab,ti OR 'online intervention':ab,ti OR 'telecommunication':ab,ti | 365961 | 21-Aug-24 |
| #11 | 'digital health':ab,ti OR 'digital medicine system':ab,ti OR 'digital behavior change':ab,ti | 7834 | 21-Aug-24 |
| #12 | 'e-mail based':ab,ti OR 'email based':ab,ti OR 'electronic mail based':ab,ti | 499 | 21-Aug-24 |
| #13 | 'telecare':ab,ti OR 'e-care':ab,ti OR 'e-consultation':ab,ti OR 'e-counselling':ab,ti OR 'remote consultation':ab,ti | 2117 | 21-Aug-24 |
| #14 | 'telehealth':ab,ti OR 'e-health':ab,ti OR 'ehealth':ab,ti OR 'electronic health':ab,ti OR 'etherap*':ab,ti OR 'e-therap*':ab,ti OR 'tele-health':ab,ti OR 'tele-care':ab,ti OR 'telecare':ab,ti OR 'telehealthcare':ab,ti OR 'tele-healthcare':ab,ti OR 'tele-homecare':ab,ti OR 'telemedicine':ab,ti OR 'tele-medicine':ab,ti OR 'telemental health':ab,ti OR 'telerehabilitation':ab,ti OR 'tele-rehabilitation':ab,ti OR 'telepsychiatry':ab,ti OR 'teletherap*':ab,ti OR 'therapy, computer-assisted':ab,ti OR 'connected health':ab,ti | 109033 | 21-Aug-24 |
| #15 | 'telemetry':ab,ti OR 'telemonitor':ab,ti OR 'telemonitoring':ab,ti OR 'tele-monitoring':ab,ti OR 'remote sensing technology':ab,ti OR 'smartphone technology':ab,ti OR 'virtual reality':ab,ti | 41520 | 21-Aug-24 |
| #16 | 'social media':ab,ti OR 'wechat':ab,ti OR 'twitter':ab,ti OR 'microblog':ab,ti OR 'weibo':ab,ti | 45996 | 21-Aug-24 |
| #17 | #6 OR #7 OR #8 OR #9 OR #10 OR #11 OR #12 OR #13 OR #14 OR #15 OR #16 | 676682 | 21-Aug-24 |
| #18 | 'pregnancy':ab,ti | 648275 | 21-Aug-24 |
| #19 | #17 AND #18 | 11712 | 21-Aug-24 |
| #20 | #19 AND 'randomized controlled trial'/de | 1101 | 21-Aug-24 |

**3.Searching results of Web of science**

| Entitlements | # | Search Query | Database | Results | Date Run |
| --- | --- | --- | --- | --- | --- |
| - WOS.IC: 1993 to 2024 - WOS.CCR: 1985 to 2024 - WOS.SCI: 1999 to 2024 | 1 | TS=((Phone) Or (mobile phone) Or (cell phone) Or (cellular phone) Or (smart phone) Or (telephone) Or (iphone) Or (Ipad) Or (Ipod) Or (iso) Or (android) Or (Computers) Or (tablet)) | Web of Science Core Collection | 709111 | Wed Aug 21 2024 22:41:52 GMT+0800 |
| - WOS.IC: 1993 to 2024 - WOS.CCR: 1985 to 2024 - WOS.SCI: 1999 to 2024 | 2 | TS=(intervention) | Web of Science Core Collection | 1166902 | Wed Aug 21 2024 22:43:06 GMT+0800 |
| - WOS.IC: 1993 to 2024 - WOS.CCR: 1985 to 2024 - WOS.SCI: 1999 to 2024 | 3 | #2 AND #1 | Web of Science Core Collection | 43145 | Wed Aug 21 2024 22:43:17 GMT+0800 |
| - WOS.IC: 1993 to 2024 - WOS.CCR: 1985 to 2024 - WOS.SCI: 1999 to 2024 | 4 | (TS=(application*)) or TS=(app*) | Web of Science Core Collection | 12590199 | Wed Aug 21 2024 22:43:58 GMT+0800 |
| - WOS.IC: 1993 to 2024 - WOS.CCR: 1985 to 2024 - WOS.SCI: 1999 to 2024 | 5 | #1 AND #4 | Web of Science Core Collection | 345512 | Wed Aug 21 2024 22:44:48 GMT+0800 |
| - WOS.IC: 1993 to 2024 - WOS.CCR: 1985 to 2024 - WOS.SCI: 1999 to 2024 | 6 | #3 OR #5 | Web of Science Core Collection | 368797 | Wed Aug 21 2024 22:44:58 GMT+0800 |
| - WOS.IC: 1993 to 2024 - WOS.CCR: 1985 to 2024 - WOS.SCI: 1999 to 2024 | 7 | (((TS=(wearable device)) OR TS=(wearable technology)) OR TS=(pORtable device)) OR TS=(mobile device) | Web of Science Core Collection | 102530 | Wed Aug 21 2024 22:47:07 GMT+0800 |
| - WOS.IC: 1993 to 2024 - WOS.CCR: 1985 to 2024 - WOS.SCI: 1999 to 2024 | 8 | ((TS=(mobile health)) OR TS=(mobile health technology)) OR TS=(mobile tech) | Web of Science Core Collection | 31520 | Wed Aug 21 2024 22:47:24 GMT+0800 |
| - WOS.IC: 1993 to 2024 - WOS.CCR: 1985 to 2024 - WOS.SCI: 1999 to 2024 | 9 | TS=((((((text message) OR (multimedia)) OR (multi-media)) OR (SMS)) OR (web-based)) OR (internet based)) | Web of Science Core Collection | 192465 | Wed Aug 21 2024 22:47:49 GMT+0800 |
| - WOS.IC: 1993 to 2024 - WOS.CCR: 1985 to 2024 - WOS.SCI: 1999 to 2024 | 10 | TS=(((((online) OR (online communication)) OR (on-line communication)) OR (online intervention)) OR (telecommunication)) | Web of Science Core Collection | 373885 | Wed Aug 21 2024 22:48:48 GMT+0800 |
| - WOS.IC: 1993 to 2024 - WOS.CCR: 1985 to 2024 - WOS.SCI: 1999 to 2024 | 11 | TS=(((digital health) OR (digital medicine system)) OR (digital behaviOR change)) | Web of Science Core Collection | 38903 | Wed Aug 21 2024 22:49:09 GMT+0800 |
| - WOS.IC: 1993 to 2024 - WOS.CCR: 1985 to 2024 - WOS.SCI: 1999 to 2024 | 12 | TS=(((( (e-mail based)) OR (email-based)) OR (email based)) OR (electronic mail based)) | Web of Science Core Collection | 10837 | Wed Aug 21 2024 22:49:25 GMT+0800 |
| - WOS.IC: 1993 to 2024 - WOS.CCR: 1985 to 2024 - WOS.SCI: 1999 to 2024 | 13 | TS=((((e-care) OR (e-consultation)) OR (E-counselling)) OR (remote consultation)) | Web of Science Core Collection | 3190 | Wed Aug 21 2024 22:49:43 GMT+0800 |
| - WOS.IC: 1993 to 2024 - WOS.CCR: 1985 to 2024 - WOS.SCI: 1999 to 2024 | 14 | TS=((((((((((((((((((((((e-health) OR (ehealth)) OR (eHealth)) OR (electronic health)) OR (eTherap*)) OR (e-Therap*)) OR (tele-health)) OR (telehealth)) OR (tele-care)) OR (telecare)) OR (telehealthcare)) OR (tele-healthcare)) OR (tele-homecare)) OR (telemedicine)) OR (tele-medicine)) OR (telemental health)) OR (Telerehabilitation)) OR (tele-rehabilitation)) OR (telepsychiatry)) OR (teletherap*)) OR (Therapy, Computer-Assisted)) OR (Connected Health)) | Web of Science Core Collection | 149689 | Wed Aug 21 2024 22:49:58 GMT+0800 |
| - WOS.IC: 1993 to 2024 - WOS.CCR: 1985 to 2024 - WOS.SCI: 1999 to 2024 | 15 | TS=(((((((telemetry) OR (telemonitOR)) OR (telemonitORing)) OR (tele-monitORing)) OR (Remote Sensing Technology)) OR (smartphone technology)) OR (virtual reality)) | Web of Science Core Collection | 78724 | Wed Aug 21 2024 22:50:16 GMT+0800 |
| - WOS.IC: 1993 to 2024 - WOS.CCR: 1985 to 2024 - WOS.SCI: 1999 to 2024 | 16 | TS=(((((facebook) OR (wechat)) OR (twitter)) OR (microblog)) OR (weibo)) | Web of Science Core Collection | 22056 | Wed Aug 21 2024 22:50:27 GMT+0800 |
| - WOS.IC: 1993 to 2024 - WOS.CCR: 1985 to 2024 - WOS.SCI: 1999 to 2024 | 17 | #6 OR #7 OR #8 OR #9 OR #10 OR #11 OR #12 OR #13 OR #14 OR #15 OR #16 | Web of Science Core Collection | 1201847 | Wed Aug 21 2024 22:50:39 GMT+0800 |
| - WOS.IC: 1993 to 2024 - WOS.CCR: 1985 to 2024 - WOS.SCI: 1999 to 2024 | 18 | TS=(pregnancy) | Web of Science Core Collection | 396666 | Wed Aug 21 2024 22:50:54 GMT+0800 |
| - WOS.IC: 1993 to 2024 - WOS.CCR: 1985 to 2024 - WOS.SCI: 1999 to 2024 | 19 | #17 AND #18 | Web of Science Core Collection | 12347 | Wed Aug 21 2024 22:51:04 GMT+0800 |
| - WOS.IC: 1993 to 2024 - WOS.CCR: 1985 to 2024 - WOS.SCI: 1999 to 2024 | 20 | TS= clinical trial* OR TS=research design OR TS=comparative stud* OR TS=evaluation stud* OR TS=controlled trial* OR TS=follow-up stud* OR TS=prospective stud* OR TS=random* OR TS=placebo* OR TS=(single blind*) OR TS=(double blind*) | Web of Science Core Collection | 5042052 | Wed Aug 21 2024 22:51:31 GMT+0800 |
| - WOS.IC: 1993 to 2024 - WOS.CCR: 1985 to 2024 - WOS.SCI: 1999 to 2024 | 21 | #19 AND #20 | Web of Science Core Collection | 5328 | Wed Aug 21 2024 22:51:46 GMT+0800 |

**4.Searching results of Cochrane library**

#1 ((intervention)):ti,ab,kw 566301

#2 (Phone) Or (mobile phone) Or (cell phone) Or (cellular phone) Or (smart phone) Or (telephone) Or (iphone) Or (Ipad) Or (Ipod) Or (iso) Or (android) Or (Computers) Or (tablet) 91112

#3 #1 AND #2 52410

#4 ((application*) OR (app*)):ti,ab,kw 538993

#5 #1 AND #4 172429

#6 #3 OR #5 202672

#7 ((mobile health) OR (mobile health technology) OR (mobile tech)):ti,ab,kw 10337

#8 ((text message) OR (multimedia) OR (multi-media) OR (SMS) OR (web-based) OR (internet based)):ti,ab,kw 25464

#9 ((online) Or (online communication) Or (on-line communication) Or (online intervention) Or (telecommunication)):ti,ab,kw 28345

#10 ((digital health) OR (digital medicine system) OR (digital behavior change)):ti,ab,kw 7468

#11 ((e-mail based) Or (email-based) Or (email based) Or (electronic mail based)):ti,ab,kw 3833

#12 ((e-care) Or (e-consultation) Or (E-counselling) Or (remote consultation)):ti,ab,kw 995

#13 ((e-health) Or (ehealth) Or (eHealth) Or (electronic health) Or (eTherap*) Or (e-Therap*) Or (tele-health) Or (telehealth) Or (tele-care) Or (telecare) Or (telehealthcare) Or (tele-healthcare) Or (tele-homecare) Or (telemedicine) Or (tele-medicine) Or (telemental health) Or (Telerehabilitation) Or (tele-rehabilitation) Or (telepsychiatry) Or (teletherap*) Or (Therapy, Computer-Assisted) Or (Connected Health)):ti,ab,kw 35342

#14 ((telemetry) Or (telemonitor) Or (telemonitoring) Or (tele-monitoring) Or (Remote Sensing Technology) Or (smartphone technology) Or (virtual reality)):ti,ab,kw 11002

#15 ((facebook) Or (wechat) Or (twitter) Or (microblog) Or (weibo)):ti,ab,kw 2060

#16 ((wearable device) OR (wearable technology) OR (portable device) Or (mobile device)):ti,ab,kw 4703

#17 #6 Or #7 Or #8 Or #9 Or #10 Or #11 Or #12 Or #13 Or #14 Or #15 Or #16 268785

#18 (pregnancy):ti,ab,kw 79441

#19 #17 AND #18 14841

#20 (Randomized controlled trial) OR ( controlled clinical trial) OR (Randomized) OR (Randomly) 1606146

#21 #19 AND #20 12570
