# Supplementary material for: Association of Digital Health Interventions With Maternal and Neonatal Outcomes: Systematic Review and Meta-Analysis
Source: J Med Internet Res. 2025 Mar 14;27:e66580. doi: 10.2196/66580 (PMC11953608; doi:10.2196/66580)

**Multimedia Appendix 3.** To assess a potential publication bias, funnel plots are used. Egger regression test was used to calculate the publication bias, and a funnel plot was drawn to visualize it.

Figure S1. Funnel plots (Gestational weight gain)


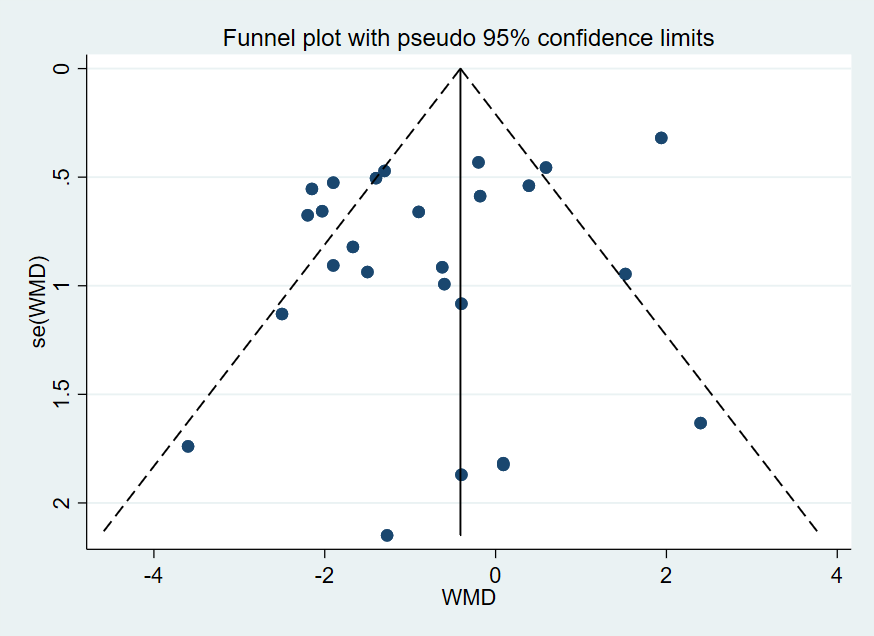


Figure S2. Funnel plots (Excessive IOM total weight gain)


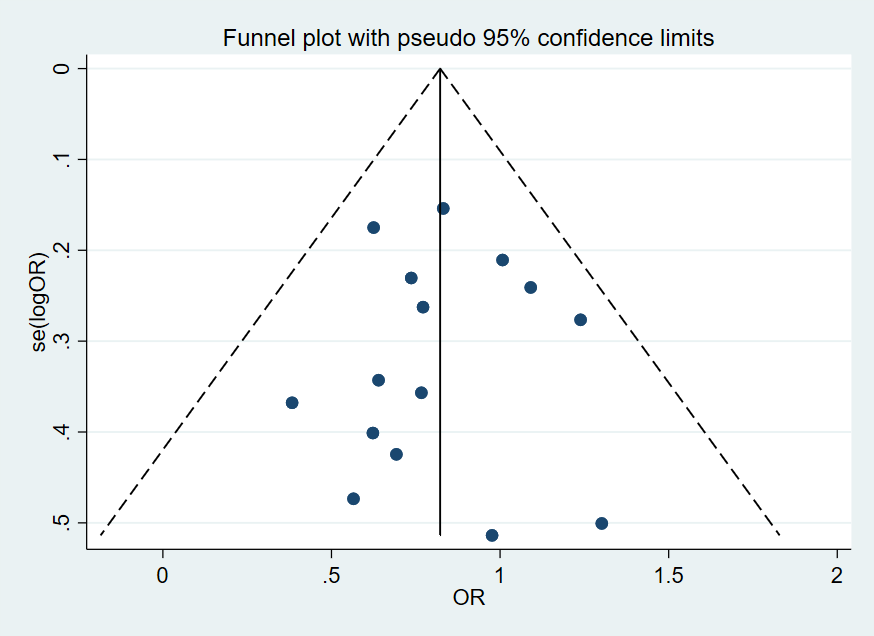


Figure S3. Funnel plots (Adequate IOM total weight gain)


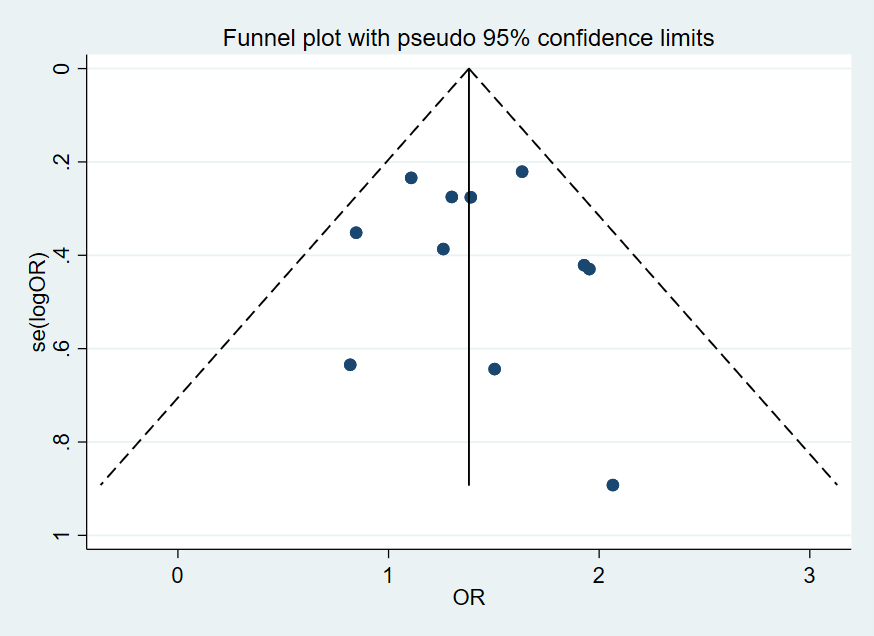


Figure S4. Funnel plots (Effect on Delivery Mode)


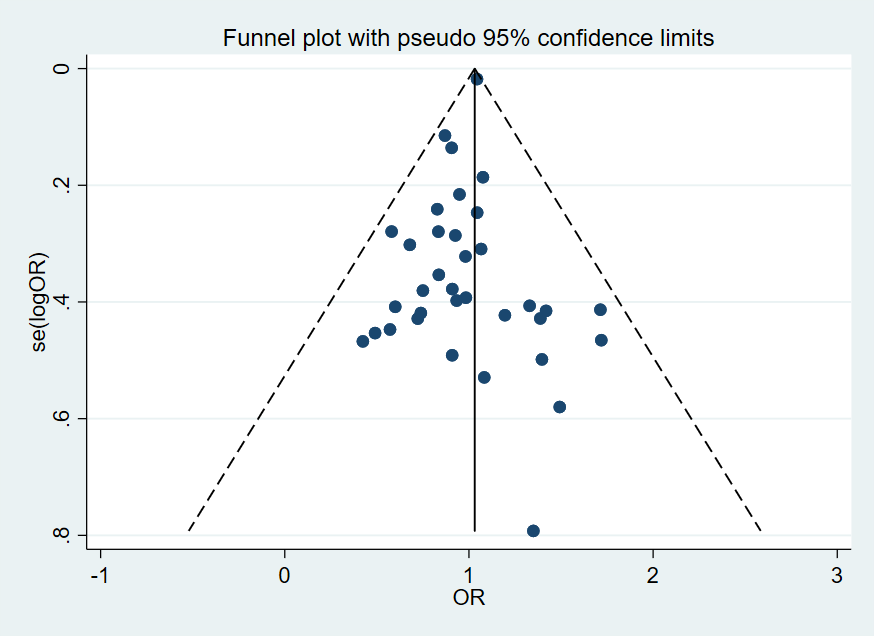


Figure S5. Funnel plots (Preterm delivery)


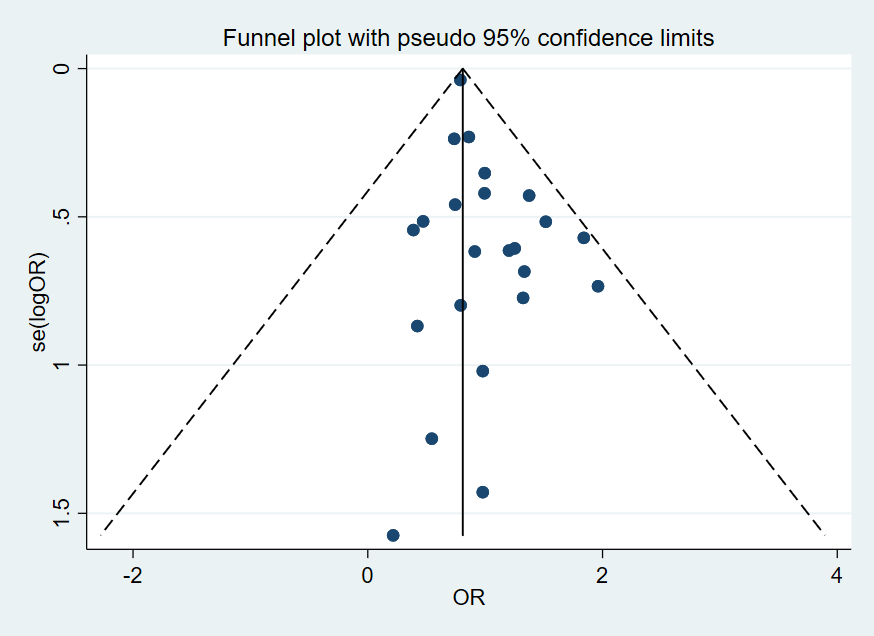


Figure S6. Funnel plots (Miscarriages)


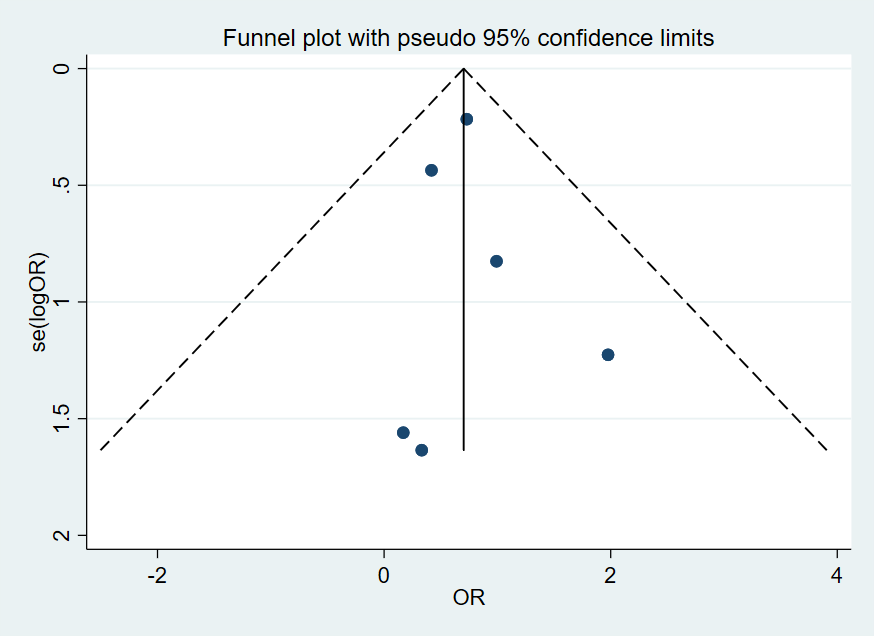


Figure S7. Funnel plots (Preterm birth)


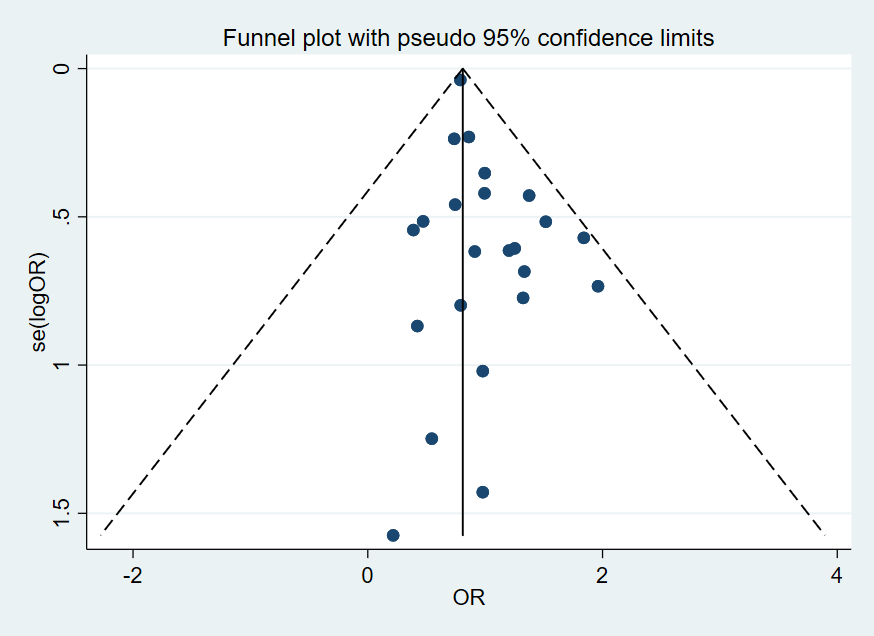

Supplement: Multimedia Appendix 3 [file jmir_v27i1e66580_app3.doc]
